# Shortcomings of reusing species interaction networks created by different sets of researchers

**DOI:** 10.1371/journal.pbio.3002068

**Published:** 2023-04-03

**Authors:** Chris Brimacombe, Korryn Bodner, Matthew Michalska-Smith, Timothée Poisot, Marie-Josée Fortin

**Affiliations:** 1 Ecology and Evolutionary Biology, University of Toronto, Toronto, Ontario, Canada; 2 MAP Centre for Urban Health Solutions, St. Michael’s Hospital, Unity Health Toronto, Toronto, Ontario, Canada; 3 Department of Ecology, Evolution and Behavior, University of Minnesota, Minneapolis, Minnesota, United States of America; 4 Department of Plant Pathology, University of Minnesota, Minneapolis, Minnesota, United States of America; 5 Département de sciences biologiques, Université de Montréal, Montréal, Québec, Canada; 6 Centre de la Science de la Biodiversité du Québec, Montréal, Québec, Canada; Estacion Biologica de Doñana CSIC, SPAIN

## Abstract

Given the requisite cost associated with observing species interactions, ecologists often reuse species interaction networks created by different sets of researchers to test their hypotheses regarding how ecological processes drive network topology. Yet, topological properties identified across these networks may not be sufficiently attributable to ecological processes alone as often assumed. Instead, much of the totality of topological differences between networks—topological heterogeneity—could be due to variations in research designs and approaches that different researchers use to create each species interaction network. To evaluate the degree to which this topological heterogeneity is present in available ecological networks, we first compared the amount of topological heterogeneity across 723 species interaction networks created by different sets of researchers with the amount quantified from non-ecological networks known to be constructed following more consistent approaches. Then, to further test whether the topological heterogeneity was due to differences in study designs, and not only to inherent variation within ecological networks, we compared the amount of topological heterogeneity between species interaction networks created by the same sets of researchers (i.e., networks from the same publication) with the amount quantified between networks that were each from a unique publication source. We found that species interaction networks are highly topologically heterogeneous: while species interaction networks from the same publication are much more topologically similar to each other than interaction networks that are from a unique publication, they still show at least twice as much heterogeneity as any category of non-ecological networks that we tested. Altogether, our findings suggest that extra care is necessary to effectively analyze species interaction networks created by different researchers, perhaps by controlling for the publication source of each network.

## Abstract 2

Researchers commonly reuse previously published species interaction networks to infer community structure and dynamics. This study reveals that these reused networks are very topologically diverse and, without proper attention to their unwanted heterogeneities, are unlikely to yield valid conclusions about ecological processes.

## Introduction

Network approaches are routinely used as a tool to analyze ecological systems [1–4]. This popularity extends to species interaction networks which model ecological communities, where nodes represent species and edges represent their corresponding species interactions [5]. Due to the effort needed to observe species and their interactions in situ, creating species interaction networks requires a tremendous amount of resources for their adequate construction [6–8]. Given this requisite effort, instead of creating their own networks, ecologists often reuse available species interaction networks which happen to be created by different sets of researchers, to test their own ecological hypotheses [9,10]. These available species interaction networks have therefore been used in many ecological studies including when determining the complexity of networks [11], identifying common topological properties across networks [12,13], and evaluating how network topology is shaped by species traits [14], environmental factors/space [7,15–17], and time [18–20].

**Fig 1:**
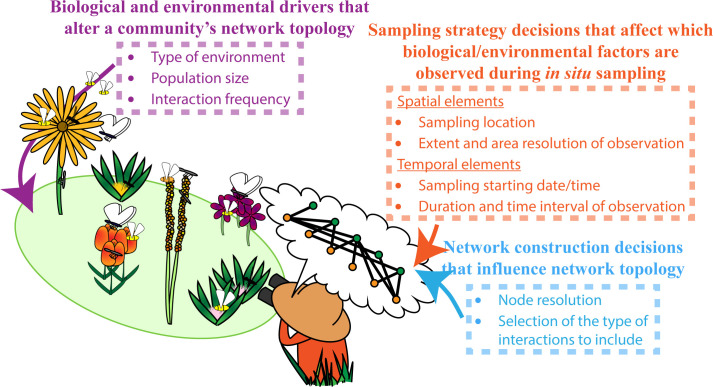
Potential sources of topological heterogeneity that influence researchers’ interpretation of a plant–pollinator community as a bipartite network. Here, the observed plant–pollinator community (green oval) is translated into a researcher’s network representation (thought bubble). Sources of topological heterogeneity between different researchers’ network interpretations of a community could be introduced from: (i) observing different biological and environmental drivers (purple text) that influence the community’s interactions, (ii) the different selected sampling strategies (orange text) that influence which biological and environmental factors are included during a researcher’s observation, and (iii) the different selected network construction methods (blue text) researchers use to design a species interaction network.

A current crux of reusing available species interaction networks created by different sets of researchers, however, is the unwanted topological differences that can exist due to the lack of consistency in the way ecological systems are translated into networks by different sets of researchers [17,21–27] (Fig 1). While some criticisms related to this issue had been raised in the 1980s/90s, e.g., Paine (1988) [28] and Polis (1991) [29], with the increasing availability to the internet and growth in computational power, a renewed interest in networks was sparked, and many of these concerns were overlooked [30].

**Fig 2:**
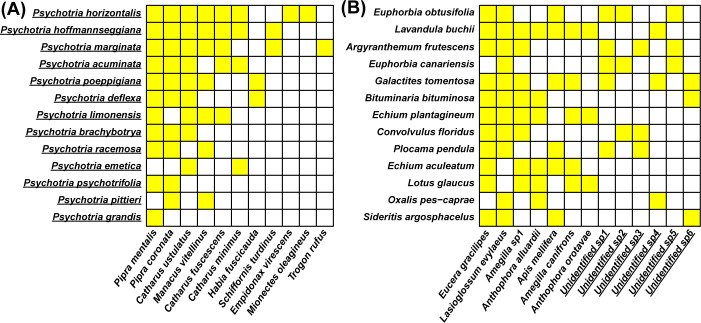
Matrix representations of 2 bipartite species interaction networks from www.web-of-life.es; an open species interaction network database. Yellow boxes in each matrix indicate the presence of an interaction between species at the corresponding row (plants) and column (animals). **(A)** Seed-dispersal network from Poulin and colleagues (1999) [41], where all plant species (underlined) are from the genus *Psychotria*. **(B)** Subset of the plant–pollinator network from Stald (2003) [42], which includes a large number of unidentified pollinator species (underlined; 34 of the 54 total pollinator species (not all shown here) in the whole network).

In order to effectively reflect on these criticisms and overall problems that occur when reusing species interaction networks created by different sets of researchers, we thought it necessary to first have a vocabulary to do so. As such, we introduce a framework that partitions how the totality of these topological differences—topological heterogeneity—between species interaction networks created by different sets of researchers can originate, by broadly organizing its sources into 3 classes (Table 1): biological and environmental drivers, sampling strategies, and network construction methods. The biological and environmental drivers class consists of sources of topological heterogeneity that arise from the different (a)biotic conditions that shape species and their interactions across different communities. For example, abiotic conditions including temperature can influence whether a species persists as well as modify their interactions [31]. Likewise, biotic drivers such as population sizes can influence both the existence and strength of interactions [32,33]. The sampling strategies class consists of sources of topological heterogeneity that arise from the different study design decisions made by researchers when observing species and their interactions and determines which effects from (a)biotic factors are included in the network, e.g., how larger sampling area and larger sampling time can capture greater environmental factors. The network construction methods class consists of sources of topological heterogeneity that are introduced via the different decisions made by researchers when constructing each network, e.g., only using plant species from a single genus (Fig 2A) or including unidentified species in the network (Fig 2B). In combination, these classes of topological heterogeneity make it incredibly difficult to decipher which topological properties in species interaction networks might be due to the ecological process of interest rather than due to unwanted sources of heterogeneity.

**Table 1:** Classes of topological heterogeneity that influence species interaction networks, some sources of this topological heterogeneity, a description of the source, and references.

Classes of heterogeneity	Source	Description of source	Example references
Biological and environmental drivers	Type of environment	Abiotic conditions influence topology (e.g., plant–pollinator networks are structured differently across a temperature gradient)	Welti and Joern (2015) [31], Pellissier and colleagues (2018) [7]
	Population sizes	Species abundances influence probability of interaction (e.g., topological differences in communities with high abundances vs. low abundances of all species)	Vázquez and colleagues (2007) [32], Vázquez and colleagues (2009) [33]
	Interaction frequencies	Number of interaction events an organism has influences the probability interactions are recorded in a network (e.g., topological differences in networks when cryptic interactions are included)	Pringle and Hutchinson (2020) [30], Pringle (2020) [34]
Sampling strategies	Temporal elements of study	The duration and time interval of observation used to characterize a community influence its representation as a network (e.g., topological differences when networks are constructed from data collected over a day vs. week)	CaraDonna and Waser (2020) [18], Schwarz and colleagues (2020) [19], CaraDonna and colleagues (2021) [20]
	Spatial elements of study	The extent and area resolution of observation used to characterize a community influence its representation as a network (e.g., topological differences when networks are constructed from data collected in a patch vs. a forest)	Galiana and colleagues (2018) [35], Galiana and colleagues (2022) [36]
Network construction methods	Selection of interaction types	Interaction types differently influence communities (e.g., topological differences between mutualistic and antagonistic systems)	Thébault and Fontaine (2010) [37], Allesina and Tang (2012) [38]
	Node resolution	Organismal classification and targeted species influences topology (e.g., topological differences when nodes represent species vs. genus or when nodes additionally represent ontogentic stages)	Hemprich-Bennett and colleagues (2021) [39], Bodner and colleagues (2022) [40]

Structural differences between species interaction networks are not problematic per se since topological heterogeneity is necessary for determining drivers of that topology. However, large amounts of topological heterogeneity between networks created by different sets of researchers may be indicative of networks that lack commensurability [43]. While some studies attempt to control for inconsistencies in the way species interaction networks are created by different researchers, e.g., controlling for sampling effort [44] or network size [45], these controls do not account for all associated unwanted topological heterogeneity when using the many different topological metrics adopted by ecologists. Hence, evaluating the amount of topological heterogeneity present in species interaction networks created by different researchers is a necessity. One approach to do this is by comparing the dispersion of network topology within species interaction networks to other real world networks that are not significantly hampered by the classes of heterogeneity listed in Table 1. If a system is accurately portrayed by its own networks, we would expect these networks to have a small amount of dispersion expressed within the metrics used to capture their topology.

As an attempt to quantify ecological topological heterogeneity, we used the largest set of bipartite networks and measured the amount of topological dispersion in (i) species interaction networks compared to non-ecological networks; and (ii) species interaction networks created by the same set of researchers (i.e., networks from the same publication) compared to the species interaction networks each a product of their own publication. We quantified differences in network topology using directed graphlet correlation distance [46], a heuristic method that measures the Euclidean distance between networks, where networks closer together are those that are more topologically similar.

To measure topological heterogeneity, we evaluated the total dispersion in directed graphlet correlation distances between networks of the same domain (defined below). As most ecological networks do not have metadata regarding the conditions under which each network was created (i.e., their associated biological and environmental drivers, sampling strategies, and network construction methods), we could not partition topological heterogeneity across the heterogeneity classes. However, as we quantified the total dispersion in non-ecological networks from different domains which are, to a large extent, not hampered by the 3 classes of heterogeneity, we could use these dispersion values to estimate the total amount of topological heterogeneity as a result of all 3 heterogeneity classes in species interaction networks. Furthermore, we compared the total dispersion of species interaction networks from the same publication to those that are not from the same publication to determine if there are topological biases due to the ways in which different sets of researchers construct networks.

## Materials and methods

### Data

A total of 3,476 bipartite networks were used in this study (see Table 2 for a description of all networks and their domains). Of the non-ecological bipartite networks included in our analysis, 1,830 were of the crime domain, 109 were of the journal domain, 245 were of the legislature domain, 172 were of the actor domain, 194 were of the sports domain, and 203 were of the microbiome domain. We classified microbiome networks as non-ecological since, among other properties, they were not built using observational data (instead, for example, by swabs and subsequent RNA sequencing) and had concrete definitions for their edges/nodes (i.e., locations on the human body where a bacterial operational taxonomic unit was found)—2 stark features that differ from species interaction networks (our ecological networks). See Aagaard and colleagues (2013) [47] for a thorough description of how patients were selected, and operational taxonomic units were sampled, which were the data we used to build the microbiome networks. Although microbiome networks could be considered ecological, we believed that their topological heterogeneity would more resemble non-ecological networks and thus grouped them accordingly. Except for sports networks that we constructed for this paper and whose data were obtained from Lahman (2021) [48], www.basketball-reference.com, and www.hockey-reference.com, all non-ecological networks were obtained from Michalska-Smith and Allesina (2019) [13]. Of the 723 species interaction networks used in this analysis (obtained from Brimacombe and colleagues (2022) [10]), 10 were ant–plant networks, 97 were host–parasite networks, 41 were plant–herbivore networks, 298 were plant–pollinator networks, and 277 were seed–dispersal networks. All networks that were included in our analysis were unweighted (i.e., interactions between nodes were binary).

**Table 2:** Description of bipartite networks used in this study.

Network domain	Node set 1	Node set 2	A connection forms when	Subgroup each network represents
Species interaction	Species	Species	A species feeds on another	Ant–plant, host–parasite, plant–herbivore, plant–pollinator, or seed–dispersal community
Actor	Actors	Film	An actor appears in a film	Action, adventure, animation, comedy, crime, documentary, drama, family, fantasy, foreign, history, horror, music, mystery, romance, science fiction, thriller, tv movie, war, or western movie genre across a number of years
Crime	Type of crimes	A city’s neighborhoods	A crime occurs in a city’s neighborhood	Chicago, Denver, Minneapolis, San Francisco, or Washington of a given day in 2016
Journal	Authors	Academic journals	An author publishes in an academic journal	All authors that published in one of The American Naturalist, Ecography, Ecological Application, Ecological Monographs, Ecology, Ecology Letters, Journal of Animal Ecology, Journal of Applied Ecology, Journal of Ecology, or Oikos, and all other academic journals within a given year (2006–2016)
Legislature	Legislators	Bills	A legislator votes positively for a bill	The US House, the US Senate, the UN General Assembly, or the European Parliament of a given year (1941–2016)
Microbiome	Bacteria operational taxonomic unit (OTU)	Locations on a human patient	A bacteria OTU is found on patient’s site	A single patient
Sports	Athletes	Teams	An athlete plays for a team	The NBA, NHL, or MLB, of a given season (1950–2020)

All bipartite networks were connected and had at least 5 nodes in either disjoint sets of nodes.

We included non-ecological systems for comparison in our study given the strict definitions used to define their systems, thereby eliminating much of the biological and environmental drivers, sampling strategies, and network construction methods classes of heterogeneity that can strongly influence the topology of species interaction networks created by different sets of researchers. Here, “strict” refers to the high likelihood that the data for these non-ecological systems were recorded consistently using such definitions that their respective nodes/edges would more accurately and precisely reflect their intended purpose when implemented as a network, as compared to species interaction networks. Furthermore, we either built each non-ecological network ourselves (i.e., sports networks), or used those previously built by us (i.e., all non-ecological networks other than sports were obtained from Michalska-Smith and Allesina (2019) [13]), thus ensuring appropriate data were used to build each non-ecological network. Indeed, the data used to build these networks came from specific databases for each domain (or subgroup within each domain, where subgroup refers to the different categories of a domain that networks represented, see Table 2). Moreover, as the data for each domain/subgroup of non-ecological networks came from the same database, if any class of heterogeneity were to influence their topology, the resulting heterogeneity would at least be consistent, and thereby reduce potential dispersion in measured topological heterogeneity. While undoubtedly the 3 classes of heterogeneity still influence non-ecological networks, for example, due to the misidentification of nodes, we expected that these classes would be significantly less influential than those within species interaction networks. In particular, we expected large amounts of topological heterogeneity in available species interaction networks created by different sets of researchers resulted from the inconsistent ways ecological communities were translated into networks by the different sets of researchers. We expected this would have introduced inconsistent topology across species interaction networks thereby increasing the dispersion in measured topological heterogeneity.

To avoid extremely small bipartite networks that may bias our results [13], we only included networks that had at least 5 nodes in either disjoint sets of nodes, e.g., we required at least 5 pollinator and 5 plant species in a plant–pollinator network. Additionally, only the giant component of each network was used (i.e., the largest connected component of a graph), given that it is unclear how to appropriately analyze disconnected networks.

### Directed graphlet correlation distance (DGCD)

In ecology, the most adopted subgraph technique is based on motifs. Generally, for a graph *G* composed of a set of nodes *V* and a set of links *L*, denoted as *G*(*V*,*L*); a motif of *G* is a subgraph *G’*(*V’*,*L’*) with a subset of nodes *V’* from *V* where any edges linking the nodes of *V’* found in *V* are contained in *L’* [49,50]. As differences in network structure are measured by which motifs are under-/overrepresented in the real network compared to a chosen network null model [46,51], like many statistical analyses, the results from the motif analysis depend on the choice of null model. As a consequence, motifs have been cited for possibly relying on ill-posed null models as a basis for significance testing [52].

**Fig 3:**
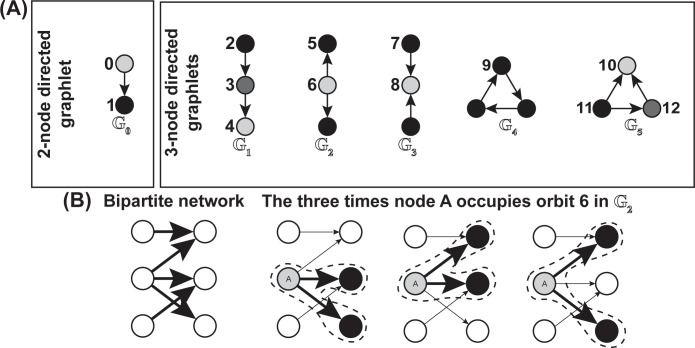
**(A)** The 6 directed graphlets (*G*_*i*_) consisting of 2 to 3 nodes and their respective orbits (i.e., the corresponding 13 numerically labeled node positions). Each unique shade in a single graphlet corresponds to a unique orbit in that graphlet. Note that for the directed bipartite networks used in this study, only graphlets *G*_0_, *G*_2_ and *G*_3_ appear. **(B)** An example calculation of the number of times node A of a directed bipartite network occupies orbit 6, where dashed lines indicate the location of *G*_2_.

To overcome the null model limitation, we instead adopted the subgraph technique of directed graphlet correlation distance (DGCD) [53] to characterize the topological differences between networks. Generally, DGCD evaluates network pairwise dissimilarity without relying on a network null model and does so by quantifying differences in the associations between the appearance of directed graphlets (Fig 3A) within a given network to those of another network.

Formally, graphlets are the induced subgraphs *G’*(*V’*,*L’*), consisting of a subset of nodes *V’* from *V* where all the edges linking the nodes of *V’* found in *V* are in the set *L’*. Within graphlets, nodes are often indistinguishable from one another. Take for example the graphlet *G*_2_ in Fig 3A: in this case, both black nodes in this graphlet are indistinguishable, and thus form an automorphism orbit—simply orbit—of a graphlet. For this reason, there is only 2 orbits within *G*_2_ labeled 5 and 6.

Generally, the DGCD relies on the directed graphlet correlation matrix (DGCM) of each network that contains Spearman’s correlations between the number of times nodes appear as particular orbits with the number of times nodes appear as all other orbits within the given network (see Fig 3B for an example count of orbit 6 for a particular node of a bipartite network). For example, the Spearman’s correlation between orbits 1 and 6 represented in a DGCM is calculated by taking the Spearman’s correlation between: (i) a vector where each index corresponds to a specific node and the entry of that index would be the number of times that node appeared as orbit 1; and (ii) same as (i) except for orbit 6. Thus, when using all 13 orbits, DGCMs were symmetric 13×13 matrices containing the respective Spearman’s correlations between the appearances of all 13 orbits within a network. Using the DGCMs, the pairwise DGCD was evaluated by measuring the pairwise Euclidean distances between all networks. See Eq (1) for a single pairwise DGCD measure between networks *K*_*i*_ and *K*_*j*_ using the 13 orbits from Fig 3A (termed DGCD-13 since it uses 13 orbits) and S1 Appendix Section S1.1 for an example derivation of the DGCD technique. We used DGCD in our study since recently Tantardini and colleagues (2019) [54] found that this method performs best at characterizing and distinguishing between networks of different domains.

$$\mathrm{DGCD}-13(K_{i},K_{j})=\sqrt{\sum_{n=0}^{12}\sum_{m=n+1}^{12}(\mathrm{DGCM}-13_{K_{i}}(n,m)-\mathrm{DGCM}-13_{K_{j}}(n,m){)}^{2}}$$ (1)

where $DGCM-13_{K_{i}}$ (*n*,*m*) is the directed graphlet correlation matrix-13’s value of network *K*_*i*_ for orbits *n* and *m*.

Since it is expected that networks from the same domain have similar topology, it is also expected that their DGCMs are similar, and consequently have small pairwise DGCD. Thus, when projected in visual space, networks from the same domain should be clustered together.

**Fig 4:**
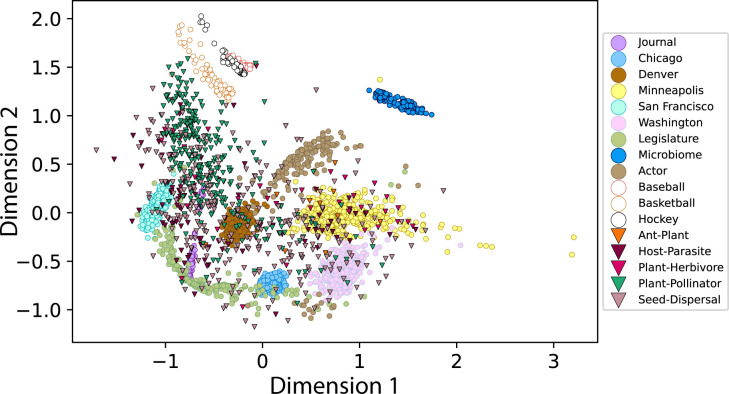
Multidimensional scaling of the pairwise directed graphlet correlation distance-13 (DGCD-13) between all bipartite networks (*n* = 3,476). Except for species interaction networks (triangles), only networks that formed clear groups in the plot are uniquely identified by color. Each point in the plot is a single network. The data and code needed to generate this figure can be found in www.osf.io/my9tv.

We calculated the pairwise DGCD-13 for all bipartite networks, where we assigned directions to the edges in the networks. Since bipartite networks are characterized by 2 sets of nodes where nodes belonging to the same set cannot have an edge, we assigned nodes belonging to 1 set to always represent a “to” direction and the other set of nodes to always represent a “from” direction in the directed edges. Simply put, this means that the DGCD-13 technique could recognize which nodes belonged to which set of nodes (e.g., which nodes belonged to the pollinator set of nodes and which nodes belonged to the plant set of nodes in a plant–pollinator network). According to these direction definitions imposed on the networks, only graphlets *G*_0_, *G*_2_, and *G*_3_ could appear although all 6 graphlets and 13 orbits were used for better visualization—specifically Fig 4—but see S1 Appendix Section S1.4 for subsequent analyses using only the 6 orbits from graphlets *G*_0_, *G*_2_, and *G*_3_, termed DGCD-6. Nevertheless, we note that the results presented in this article for DGCD-13 agree with those presented in S1 Appendix Section S1.4 using DGCD-6.

From all pairwise DGCD-13s, we measured the dispersion of network topology by calculating the mean pairwise distances between all networks of the same domain. In cases where subgroups (e.g., hockey networks) formed coherent topology that was different from their domain (e.g., the mean pairwise DGCD-13 was much smaller for hockey networks compared to all other sports networks), we instead evaluated the mean pairwise DGCD-13 for that subgroup. If the set of networks from the same domain or subgroup had small mean pairwise DGCD-13, then this would indicate that these networks have small dispersion in their topology, i.e., they are similarly structured.

Additionally, we tested whether species interaction networks created by the same set of researchers (i.e., networks sourced from the same publication) were more topologically similar than networks not sourced from the same publication, see Table C in S1 Appendix for a list of publications that provided more than a single network. Specifically, we compared the mean pairwise DGCD-13 of networks from the same publication to the mean pairwise DGCD-13 of networks that were each a product of their own publication. Given that networks constructed by the same researchers are likely more parsimonious in terms of their topology, we expected that the mean pairwise DGCD-13 between networks from the same publication were going to be smaller than networks each produced by different publications.

## Results

The pairwise DGCD-13 between all networks was projected via multidimensional scaling (MDS) [55], also commonly known as principal coordinate analysis, using the MDS function in the Scikit-learn library [56] of Python. Except for species interaction networks, only networks that formed clear clusters were uniquely colored and identified in the MDS plot (Fig 4). Most networks from the same domain occurred in the same location in the plot and were isolated from other networks’ domains in the MDS space except for species interaction, sports, and crime networks. With regards to species interaction networks, no coherent topology was observed as these networks covered all other types of non-ecological networks besides microbiome and sports networks. With regards to sports and crime networks, specific cities (i.e., the subgroups of Chicago, Denver, Minneapolis, San Francisco, and Washington) and specific sports (i.e., the subgroups of hockey, baseball, and basketball) had unique topology and formed their own respective subgroupings within the plot, and thus despite not having the same topology, there was clear topological coherence within a city’s own set of crime networks and a sports’ own set of networks. Here, subgrouping refers to networks from a specific subgroup that formed clear and unique clusters in the MDS plot. Since every network’s domain was composed of multiple different subgroups (e.g., actor networks were made from action, adventure, …, western movie genres/subgroups, Table 2), each domain could have potentially formed their own distinct subgroupings within Fig 4 if they exhibited unique substructure like crime and sports networks.

Of networks from the same domain or networks that had their own subgroupings within the MDS plot (Fig 4), species interaction networks had the largest mean pairwise DGCD-13 of 1.101—about twice as much as the set of legislation networks which was the next domain or subgrouping with the most topological dispersion (Table 3). This pattern also held when using median pairwise DGCD-13 (Table B in S1 Appendix) and so mean DGCD-13 was not significantly influenced by outliers. As well, the large variability in the size of species interaction networks did not contribute to this larger mean pairwise DGCD-13 value (Table D in S1 Appendix). Interestingly, both legislation and Minneapolis crime networks also had relatively high mean pairwise DGCD-13 (0.578 and 0.509, respectively), although legislation networks were composed of 4 subgroups that did not form subgroupings in the MDS plot (i.e., US House, US Senate, UN General Assembly, and European Parliament) which likely contributed to this larger value.

**Table 3:** Mean pairwise directed graphlet correlation distance-13 (DGCD-13) between bipartite networks from the same domain or subgrouping. Subgrouping refers to a subgroup [i.e., networks classified as the same type of network from the same domain during network construction (e.g., the Chicago networks in the crime network domain)] that formed an obvious cluster within the MDS plot (Fig 4). See Table 2 for a list of network domains and their corresponding subgroups. All species interaction networks were classified into their appropriate subgroup even though they did not form subgroupings.

Network domain	Subgrouping or subgroup	Mean pairwise DGCD-13	Number of networks
Species interaction	Ant–plant	0.794	10
	Host–parasite	1.064	97
	Plant–herbivore	1.264	41
	Plant–pollinator	0.890	298
	Seed–dispersal	1.092	277
	None^1^	1.101	723
Actor		0.414	172
Crime	Chicago	0.129	366
	Denver	0.180	366
	Minneapolis	0.509	366
	San Francisco	0.169	366
	Washington	0.305	366
Journal		0.234	109
Legislation		0.578	245
Microbiome		0.241	203
Sports	Baseball	0.200	71
	Basketball	0.459	68
	Hockey	0.330	55

^1^DGCD-13 between all species interaction networks.

Exclusively within the species interaction domain, networks from the same publication were more topologically similar, by about a factor of 2, than networks that were each a product of their own publication (0.544 and 1.134 mean pairwise DGCD-13, respectively, Table 4). This smaller dispersion within networks from the same publication was also about 32% less than the topological dispersion within the ecological subgroup that had the least topological dispersion, i.e., ant–plant (0.794 pairwise DGCD-13, Table 3). It should be noted, however, that while ant–plant networks were the least topologically heterogeneous subgroup of species interaction networks tested, this should not be generalized given that we only had a few networks available to us (*n* = 10), which were sourced from only 3 publications. Nevertheless, although networks from the same publication were generally of the same species interaction subgroup (i.e., most networks from a specific publication belonged to only one of either ant–plant, host–parasite, plant–herbivore, plant–pollinator, or seed–dispersal subgroup), networks from the same publication were more topologically similar than any single species interaction subgroup.

**Table 4:** Mean pairwise directed graphlet correlation distance-13 (DGCD-13) of bipartite species interaction networks from the same publication grouping.

Publication grouping	Mean pairwise DGCD-13	Number of networks	Number of publications
One network per publication	1.134	236	236
Multiple networks per publication	0.544^1^	487	58

^1^Calculated by taking the mean of the average pairwise DGCD-13s between networks from the same publication, weighted by the number of networks produced by each publication.

Multiple bipartite networks sourced from the same publication (i.e., networks created by the same set of researchers) are termed “multiple networks per publication” and bipartite networks sourced from publications that each produced only a single network are termed “one network per publication.” See Table C in S1 Appendix for a list of publications that provided more than 1 network and each publication’s mean pairwise DGCD-13.

## Discussion

Ecologists commonly reuse species interaction networks created by different sets of researchers to test how ecological and environmental processes shape network topology across space and time [8,9,26]. However, unwanted topological differences as a result of the different ways in which researchers translate ecological communities into networks could inhibit their commensurability [10,23,24]. When assessing the degree of topological heterogeneity, i.e., the total amount of topological differences between a group of networks, we find that species interaction networks created by different sets of researchers are extremely topologically heterogeneous—about twice the amount than the next most heterogeneous network domain tested—and that this large heterogeneity is linked to the publication source of each network. Altogether, these findings suggest that species interaction networks created by different sets of researchers can be problematic for deducing ecological topological rules since much of the topological heterogeneity is likely not due to ecological processes as is often assumed.

A general principle in statistics is that an increased sample size reduces uncertainties of estimators [57]. Armed with this principle, and the ease with which species interaction networks can be obtained from online resources [25], it may then be tempting to assume that increasing one’s data set by collecting all possible networks available alleviates any data issues. However, using the largest set of bipartite species interaction networks available (*n* = 723), we illustrate how large amounts of topological heterogeneity (via the mean pairwise DGCD-13, Table 3) and consequently uncertainty exists in the topology of species interaction networks created by different sets of researchers, confirming that more data is not always better when biases are present [57]. While some metrics, including sampling intensity and effort, have previously been used to control for biases and sources of topological heterogeneity in species interaction networks [43], these controls do not effectively account for all sources of heterogeneity (e.g., differences in node taxonomy across networks) or when using different topological metrics (e.g., modularity, nestedness). Hence, careful consideration, beyond a single metric of control, is required when deciding which networks to include in one’s analyses, so that the majority of topological differences measured between species interaction networks are a result of the ecological process-of-interest and not from confounding factors.

The large amount of topological heterogeneity in species interaction networks created by different sets of researchers likely reflects their topological uniqueness due to the distinct (a)biotic conditions each represented community experiences, the distinct sampling strategies adopted to characterize each ecological system as a network, and the distinct construction methods used to create each network (Table 1). Indeed, the large difference in the amount of topological heterogeneity between species interaction networks and non-ecological networks may be attributed to these 3 classes of topological heterogeneity given that the non-ecological networks were created in a consistent way to try to eliminate much of their influence. For example, we built non-ecological networks using data attained from consistent sampling strategies (e.g., each sampled crime network represented a specific city and day of the year in 2016) and we used consistent network construction definitions when building the networks from the data (e.g., all interactions in crime networks always represented a type of crime occurring in a city’s specific neighborhood). This is not to say that non-ecological networks are devoid of their own sources of topological heterogeneity. For example, differences in both voter sentiment across time and differences in the political landscape across space within the legislative networks (e.g., between US House and UN General Assembly networks) likely contribute some topological heterogeneity. However, the biological and environmental drivers, sampling strategies, and network construction methods classes of heterogeneity seem to be accentuated in species interaction networks created by different sets of researchers as compared to the tested non-ecological networks.

Importantly, even though biological and environmental drivers, sampling strategies, and network construction methods classes of heterogeneity are known to influence the topology of species interaction networks, they are nevertheless rarely acknowledged or appropriately controlled in ecological studies. This is especially problematic when reusing networks created by different sets of researchers since the influence of these classes are likely to vary considerably depending on the methods and approaches that different researchers use to create each network. In fact, rarely are differences in sampling strategies controlled for when reusing networks, even though sampling strategies influence network topology. For example, species interaction networks are already topologically different when constructed from observational data collected over different amounts of time [18–20], or over different amounts of area [35,36]. Furthermore, related to variations in sampling strategies, networks may also vary in their sampling sufficiency [58]. Insufficiency can occur when the sampling design does not match the biology of the community and can make networks incommensurable even when networks are built using the same sampling strategies. Moreover, differences in biological and environmental drivers that ecological communities experience are sometimes not controlled for when reusing networks, even though these drivers can influence network topology. For example, species interaction networks are already topologically different depending on the temperature each community experiences [31]. As well, despite the widespread reuse of species interaction networks created by different sets of researchers for testing ecological hypotheses, it is still relatively unknown how different network construction methods influence topology, which may also make network comparison difficult. For example, interactions in one plant–pollinator network can represent a pollinator touching a plant and in another network represent pollen of a plant being found on a pollinator [59], or networks may or may not contain pollinators which are commensals or parasitic to plants [60]. Thus, without care and appropriate control of the topological differences from the 3 heterogeneity classes, one is liable to find erroneous relationships when reusing species interaction networks created by different sets of researchers [45,61].

All is not lost when reusing species interaction networks created by different sets of researchers, as one approach to avoid a large amount of the topological heterogeneity may be to attempt to control for the publication source of each network. While we found a large amount of topological heterogeneity between all species interaction networks, we also found that networks created by the same set of researchers (i.e., networks from the same publication) were more topologically similar to each other (Table 4). Interestingly, we also found that networks from the same publication were more topologically similar than networks from any species interaction subgroup (i.e., networks belonging only to either ant–plant, host–parasite, plant–herbivore, plant–pollinator, or seed–dispersal). Consequently, it appears that publication has an even greater impact on the topology of species interaction networks than biological processes alone. This may occur since researchers of a given publication generally construct networks under parsimonious conditions [10], for example, by observing and characterizing ecological communities across the same time duration (e.g., Trøjelsgaard and colleagues (2015) [62]) or by classifying nodes across networks using the same protocol (e.g., Pereira Martins and colleagues (2020) [63]), and thus inadvertently control for many sources of topological heterogeneity. Of course, biological effects are likely influencing the topology of all networks but they can be more easily obscured when analyzing networks across publications. It is then likely that controlling for the effect of publication can reduce unwanted topological heterogeneity between networks. We do, however, strongly caution those that only attempt to account for the publication source of each reused network. Similar to how different network metrics are sensitive to different amounts of sampling sufficiency [58], the strong similarity between networks from the same publication may be more or less relevant when investigating network structure using other metrics.

Although most researchers do not originally intend for their networks to be reused and compared to other networks, often they are included in meta-analysis type studies if they are made freely available. Original authors of networks can improve the scientific utility of their networks by providing other researchers with information about how they were constructed [26]. In particular, by providing detailed network metadata, including information on relevant biological and environmental drivers, sampling strategies, and network construction methods, authors of the networks can help others understand the specific conditions under which each network was created. Additionally, given the recent developments of composite methods designed to estimate sampling sufficiency for ecological networks (e.g., Casas and colleagues (2018) [58]), authors of species interaction networks could also calculate this metric or provide the information to do so to check if communities have been sufficiently sampled. Then, beyond controlling for sources of topological heterogeneity (e.g., node taxonomy), researchers reusing these networks could also control for sampling sufficiency which is another means to improve network commensurability. Given appropriate metadata, researchers could also study how each class of heterogeneity influences the topology of species interaction networks, rather than the totality of topological heterogeneity as we have done here.

Nevertheless, as users of species interaction networks that happen to be constructed by different sets of researchers, the onus is on us to know the limitations of our data and to ensure that they effectively represent the systems in the corresponding models we use [64]. Given that all species interaction networks are models and are thus subject to imperfections (e.g., Pringle and Hutchinson (2020) [30]; Thomson (2021) [65]), we should be aware of their overall shortcomings and attempt to correct for them, especially since our findings are often used to inform policy aimed at conserving ecological systems.

### Caveats

A limitation in our analyses was the use of small species interaction networks (e.g., <100 nodes). Since networks with a small number of nodes and edges are generally more difficult to classify than larger networks [46], we perhaps inadvertently increased the perceived topological heterogeneity of species interaction networks as compared to some of the non-ecological networks. Regardless, the crime networks we used were of similar size to species interaction networks (Table A in S1 Appendix), but were less topologically heterogeneous (Table 3 and Fig 4). Clearly then, it was still possible to find topological consistency even in small networks, but less so when networks were both small and ecological. This suggests that the topological heterogeneity in species interaction networks created by different sets of researchers was due to more than just the difficulty of classifying small networks, but likely also from biological and environmental drivers, sampling strategies, and network construction methods classes of heterogeneity, which reused networks created by different sets of researchers are especially prone to. Importantly, this same problem of using small networks is also relevant when applying any other types of metrics to ecological networks, e.g., nestedness and modularity.

Although we generally failed to find pervasive and coherent topology within species interaction networks created by different sets of researchers, we highlight that our results do not necessarily invalidate patterns others have found (e.g., high nestedness in plant–animal networks [66]). Instead, these patterns are perhaps true under strict conditions, such as controlling for the unwanted differences in topology between studies when reusing their networks.

## Conclusion

Species interaction networks created by different sets of researchers likely suffer from comparison problems due to many sources of topological heterogeneity, i.e., via biological and environmental drivers, sampling strategies, or network construction methods classes of heterogeneity. Quantitatively, our findings show that these species interaction networks are remarkably topologically diverse and that we should be especially careful when reusing this source of data for deducing rules of community assembly, perhaps by controlling for the publication source of each network.

## Supporting information

S1 Appendix Detailed and additional methods, supplementary figures, and tables. (PDF)

## References

[1] Blüthgen N. Why network analysis is often disconnected from community ecology: A critique and an ecologist’s guide. Basic Appl Ecol. 2010;11(3):185–195.

[2] Poisot T, Stouffer DB, Kéfi S. Describe, understand and predict: Why do we need networks in ecology? Funct Ecol. 2016;30(12):1878–1882.

[3] Delmas E, Besson M, Brice M-H, Burkle LA, Dalla Riva GV, Fortin M-J, et al. Analysing ecological networks of species interactions. Biol Rev. 2019;94(1):16–36. doi: 10.1111/brv.12433 29923657

[4] Fortin M-J, Dale MRT, Brimacombe C. Network ecology in dynamic landscapes. Proc R Soc B. 1949;2021(288):20201889. 10.1098/rspb.2020.1889 PMC8080002 33906397

[5] Dormann CF, Fründ J, Schaefer HM. Identifying causes of patterns in ecological networks: Opportunities and limitations. Annu Rev Ecol Evol Syst. 2017;48(1):559–584.

[6] Jordano P. Sampling networks of ecological interactions. Funct Ecol. 2016;30(12):1883–1893.

[7] Pellissier L, Albouy C, Bascompte J, Farwig N, Graham C, Loreau M, et al. Comparing species interaction networks along environmental gradients. Biol Rev. 2018;93(2):785–800. doi: 10.1111/brv.12366 28941124

[8] McLeod A, Leroux SJ, Gravel D, Chu C, Cirtwill AR, Fortin M-J, et al. Sampling and asymptotic network properties of spatial multi-trophic networks. Oikos. 2021;130(12):2250–2259.

[9] Poisot T, Bergeron G, Cazelles K, Dallas T, Gravel D, MacDonald A, et al. Global knowledge gaps in species interaction networks data. J Biogeogr. 2021;48(7):1552–1563.

[10] Brimacombe C, Bodner K, Michalska-Smith MJ, Gravel D, Fortin M-J. No strong evidence that modularity, specialization, or nestedness are linked to seasonal climatic variability in bipartite networks on a global scale. Glob Ecol Biogeogr. 2022;31(12):2510–2523.

[11] Strydom T, Dalla Riva GV, Poisot T. SVD entropy reveals the high complexity of ecological networks. Front Ecol Evol. 2021;9:1–10.

[12] Mora BB, Gravel D, Gilarranz LJ, Poisot T, Stouffer DB. Identifying a common backbone of interactions underlying food webs from different ecosystems. Nat Commun. 2018;9(1):1–8. 29973596 10.1038/s41467-018-05056-0 PMC6031633

[13] Michalska-Smith MJ, Allesina S. Telling ecological networks apart by their structure: A computational challenge. PLoS Comput Biol. 2019;15(6):1–13. doi: 10.1371/journal.pcbi.1007076 31246974 PMC6597030

[14] Dalsgaard B, Maruyama PK, Sonne J, Hansen K, Zanata TB, Abrahamczyk S, et al. The influence of biogeographical and evolutionary histories on morphological trait-matching and resource specialization in mutualistic hummingbird-plant networks. Funct Ecol. 2021;35(5):1120–1133.

[15] Olesen JM, Jordano P. Geographic patterns in plant-pollinator mutualistic networks. Ecology. 2002;83(9):2416–2424.

[16] Dalsgaard B, Schleuning M, Maruyama PK, Dehling DM, Sonne J, Vizentin-Bugoni J, et al. Opposed latitudinal patterns of network-derived and dietary specialization in avian plant-frugivore interaction systems. Ecography. 2017;40(12):1395–1401.

[17] Doré M, Fontaine C, Thébault E. Relative effects of anthropogenic pressures, climate, and sampling design on the structure of pollination networks at the global scale. Glob Chang Biol. 2021;27(6):1266–1280. doi: 10.1111/gcb.15474 33274540

[18] CaraDonna PJ, Waser NM. Temporal flexibility in the structure of plant-pollinator interaction networks. Oikos. 2020;129(9):1369–1380.

[19] Schwarz B, Vázquez DP, CaraDonna PJ, Knight TM, Benadi G, Dormann CF, et al. Temporal scale-dependence of plant-pollinator networks. Oikos. 2020;129(9):1289–1302.

[20] CaraDonna PJ, Burkle LA, Schwarz B, Resasco J, Knight TM, Benadi G, et al. Seeing through the static: The temporal dimension of plant-animal mutualistic interactions. Ecol Lett. 2021;24(1):149–161. doi: 10.1111/ele.13623 33073900

[21] Dormann CF, Fründ J, Blüthgen N, Gruber B. Indices, graphs and null models: Analyzing bipartite ecological networks. Open Ecol J. 2009;2(1):7–24.

[22] Ings TC, Montoya JM, Bascompte J, Blüthgen N, Brown L, Dormann CF, et al. Review: Ecological networks—beyond food webs. J Anim Ecol. 2009;78(1):253–269. 19120606 10.1111/j.1365-2656.2008.01460.x

[23] Gibson RH, Knott B, Eberlein T, Memmott J. Sampling method influences the structure of plant-pollinator networks. Oikos. 2011;120(6):822–831.

[24] Quintero E, Isla J, Jordano P. Methodological overview and data-merging approaches in the study of plant-frugivore interactions. Oikos. 2022;2022(2):e08379.

[25] Salim JA, Saraiva AM, Zermoglio PF, Agostini K, Wolowski M, Drucker DP, et al. Data standardization of plant-pollinator interactions. GigaScience. 2022;11:1–15. doi: 10.1093/gigascience/giac043 35639882 PMC9154084

[26] Mestre F, Gravel D, García-Callejas D, Pinto-Cruz C, Matias MG, Araújo MB. Disentangling food-web environment relationships: A review with guidelines. Basic Appl Ecol. 2022;61:102–115.

[27] Vázquez DP, Peralta G, Cagnolo L, Santos M. Ecological interaction networks. What we know, what we don’t, and why it matters. Ecol Austral. 2022;32:670–697.

[28] Paine RT. Road maps of interactions or grist for theoretical development? Ecology. 1988;69(6):1648–1654.

[29] Polis GA. Complex trophic interactions in deserts: An empirical critique of food-web theory. Am Nat. 1991;138(1):123–155.

[30] Pringle RM, Hutchinson MC. Resolving food-web structure. Annu Rev Ecol Evol Syst. 2020;51(1):55–80.

[31] Welti EAR, Joern A. Structure of trophic and mutualistic networks across broad environmental gradients. Ecol Evol. 2015;5(2):326–334. doi: 10.1002/ece3.1371 25691960 PMC4314265

[32] Vázquez DP, Melián CJ, Williams NM, Blüthgen N, Krasnov BR, Poulin R. Species abundance and asymmetric interaction strength in ecological networks. Oikos. 2007;116(7):1120–1127.

[33] Vázquez DP, Chacoff NP, Cagnolo L. Evaluating multiple determinants of the structure of plant-animal mutualistic networks. Ecology. 2009;90(8):2039–2046. doi: 10.1890/08-1837.1 19739366

[34] Pringle RM. In: Dobson A, Tilman D, Holt RD, editors. Untangling food webs. Princeton: Princeton University Press; 2020. p. 225–238.

[35] Galiana N, Lurgi M, Claramunt-López B, Fortin M-J, Leroux S, Cazelles K, et al. The spatial scaling of species interaction networks. Nat Ecol Evol. 2018;2(5):782–790. doi: 10.1038/s41559-018-0517-3 29662224

[36] Galiana N, Lurgi M, Bastazini VAG, Bosch J, Cagnolo L, Cazelles K, et al. Ecological network complexity scales with area. Nat Ecol Evol. 2022;6:307–314. doi: 10.1038/s41559-021-01644-4 35027724 PMC7614050

[37] Thébault E, Fontaine C. Stability of ecological communities and the architecture of mutualistic and trophic networks. Science. 2010;329(5993):853–856. doi: 10.1126/science.1188321 20705861

[38] Allesina S, Tang S. Stability criteria for complex ecosystems. Nature. 2012;483(7388):205–208. doi: 10.1038/nature10832 22343894

[39] Hemprich-Bennett DR, Oliveira HFM, Le Comber SC, Rossiter SJ, Clare EL. Assessing the impact of taxon resolution on network structure. Ecology. 2021;102(3):e03256. doi: 10.1002/ecy.3256 33226629

[40] Bodner K, Brimacombe C, Fortin M-J, Molnár PK. Why body size matters: How larger fish ontogeny shapes ecological network topology. Oikos. 2022;2022(3):e08569.

[41] Poulin B, Wright SJ, Lefebvre G, Calderón O. Interspecific synchrony and asynchrony in the fruiting phenologies of congeneric bird-dispersed plants in Panama. J Trop Ecol. 1999;15(2):213–227.

[42] Stald L. Struktur og dynamik i rum og tid af et bestø vningsnetvÃ¦rk på Tenerife, De Kanariske Ø er [Thesis]. Aarhus University; 2003.

[43] Brimacombe C, Bodner K, Fortin MJ. How network size strongly determines trophic specialisation: A technical comment on Luna et al. (2022). Ecol Lett. 2022;8(25):1914–1916. 10.1111/ele.14029 35610664

[44] Schleuning M, Fründ J, Klein AM, Abrahamczyk S, Alarcón R, Albrecht M, et al. Specialization of mutualistic interaction networks decreases toward tropical latitudes. Curr Biol. 2012;22(20):1925–1931. doi: 10.1016/j.cub.2012.08.015 22981771

[45] Morris RJ, Gripenberg S, Lewis OT, Roslin T. Antagonistic interaction networks are structured independently of latitude and host guild. Ecol Lett. 2014;17(3):340–349. doi: 10.1111/ele.12235 24354432 PMC4262010

[46] Yaveroğlu ÖN, Malod-Dognin N, Davis D, Levnajic Z, Janjic V, Karapandza R, et al. Revealing the hidden language of complex networks. Sci Rep. 2014;4(1):1–9. doi: 10.1038/srep04547 24686408 PMC3971399

[47] Aagaard K, Petrosino J, Keitel W, Watson M, Katancik J, Garcia N, et al. The Human Microbiome Project strategy for comprehensive sampling of the human microbiome and why it matters. FASEB J. 2013;27(3):1012–1022. doi: 10.1096/fj.12-220806 23165986 PMC3574278

[48] Lahman S. Lahman’s Baseball Database; 2021. Available from: seanlahman.com/baseball-archive/statistics/.

[49] Milo R, Shen-Orr S, Itzkovitz S, Kashtan N, Chklovskii D, Alon U. Network motifs: Simple building blocks of complex networks. Science. 2002;298(5594):824–827. doi: 10.1126/science.298.5594.824 12399590

[50] Stouffer DB, Camacho J, Jiang W, Nunes Amaral LA. Evidence for the existence of a robust pattern of prey selection in food webs. Proc R Soc B Biol Sci. 2007;274(1621):1931–1940. doi: 10.1098/rspb.2007.0571 17567558 PMC2275185

[51] Pržulj N, Kuchaiev O, Stevanović A, Hayes W. Geometric evolutionary dynamics of protein interaction networks. In: Biocomputing 2010. World Scientific; 2010. p. 178–189. doi: 10.1142/9789814295291_0020 19908370

[52] Artzy-Randrup Y, Fleishman SJ, Ben-Tal N, Stone L. Comment on “Network motifs: Simple building blocks of complex networks” and “Superfamilies of evolved and designed networks”. Science. 2004;305(5687):1107–1107. 10.1126/science.1099334 15326338

[53] Sarajlić A, Malod-Dognin N, Yaveroğlu ÖN, Pržulj N. Graphlet-based characterization of directed networks. Sci Rep. 2016;6(1):1–14. 27734973 10.1038/srep35098 PMC5062067

[54] Tantardini M, Ieva F, Tajoli L, Piccardi C. Comparing methods for comparing networks. Sci Rep. 2019;9(1):1–19. 31772246 10.1038/s41598-019-53708-y PMC6879644

[55] Borg I, Groenen PJF. Modern multidimensional scaling: Theory and applications. Springer Science & Business Media; 2005.

[56] Pedregosa F, Varoquaux G, Gramfort A, Michel V, Thirion B, Grisel O, et al. Scikit-learn: Machine learning in Python. J Mach Learn Res. 2011;12:2825–2830.

[57] Dietze M. Ecological forecasting. Princeton University Press; 2017. 10.1515/9781400885459

[58] Casas G, Bastazini VAG, Debastiani VJ, Pillar VD. Assessing sampling sufficiency of network metrics using bootstrap. Ecol Complex. 2018;36:268–275.

[59] Hagen M, Kissling WD, Rasmussen C, De Aguiar MAM, Brown LE, Carstensen DW, et al. Biodiversity, species interactions and ecological networks in a fragmented World. In: Jacob U, Woodward G, editors. Global change in multispecies systems Part 1. vol. 46 of Advances in Ecological Research. Academic Press; 2012. p. 89–210.

[60] Guimarães PR. The structure of ecological networks across levels of organization. Annu Rev Ecol Evol Syst. 2020;51(1):433–460.

[61] Ollerton J, Cranmer L. Latitudinal trends in plant-pollinator interactions: Are tropical plants more specialised? Oikos. 2002;98(2):340–350.

[62] Trøjelsgaard K, Jordano P, Carstensen DW, Olesen JM. Geographical variation in mutualistic networks: Similarity, turnover and partner fidelity. Proc R Soc B Biol Sci. 1802;2015(282):20142925. 10.1098/rspb.2014.2925 PMC4344161 25632001

[63] Pereira Martins L, Matos Medina A, Lewinsohn TM, Almeida-Neto M. The effect of species composition dissimilarity on plant-herbivore network structure is not consistent over time. Biotropica. 2020;52(4):664–674.

[64] Bodner K, Brimacombe C, Chenery ES, Greiner A, McLeod AM, Penk SR, et al. Ten simple rules for tackling your first mathematical models: A guide for graduate students by graduate students. PLoS Comput Biol. 2021;17(1):1–12. doi: 10.1371/journal.pcbi.1008539 33444343 PMC7808623

[65] Thomson J. Editorial: How worthwhile are pollination networks? J Pollinat Ecol. 2021;28:i–vi.

[66] Bascompte J, Jordano P. Plant-animal mutualistic networks: The architecture of biodiversity. Annu Rev Ecol Evol Syst. 2007;38(1):567–593.

