# Supplementary material for: Shortcomings of reusing species interaction networks created by different sets of researchers
Source: PLoS Biol. 2023 Apr 3;21(4):e3002068. doi: 10.1371/journal.pbio.3002068 (PMC10101633; doi:10.1371/journal.pbio.3002068)
Supplement: S1 Appendix — (PDF) [file pbio.3002068.s001.pdf]

S1 Appendix

S1.1 Directed graphlet correlation distance general information

Here, we provide a brief introduction to the directed graphlet correlation distance 6 (DGCD-6). While the same calculations are performed for DGCD-13, note that *all* graphlets with three or fewer nodes (i.e., all 6 graphlets and 13 orbits in Fig A) are used when employing DGCD-13, instead of the 3 graphlets (G_0, G_2, G_3) and 6 orbits (0, 1, 5, 6, 7, 8) when employing DGCD-6. We chose to present DGCD-6 as an example derivation as opposed to DGCD-13 to simplify derivation Figs B, C, and D in S1 Appendix Section S1.1. Specifically, the only difference between the two methods is that using DGCD-13 with bipartite networks results in (*i*) additional zero entries in orbits 2, 3, 4, 9, 10, 11, and 12 for the directed graphlet degree vector in Fig B, (*ii*) additional corresponding zero vectors for orbits 2, 3, 4, 9, 11, and 12 in Fig C, and (*iii*) similar to (*ii*) additional corresponding zero vectors for orbits 2, 3, 4, 9, 11, and 12 for the two directed graphlet correlation matrices, as well as additional squared terms when evaluating the DGCD equation in Fig D.

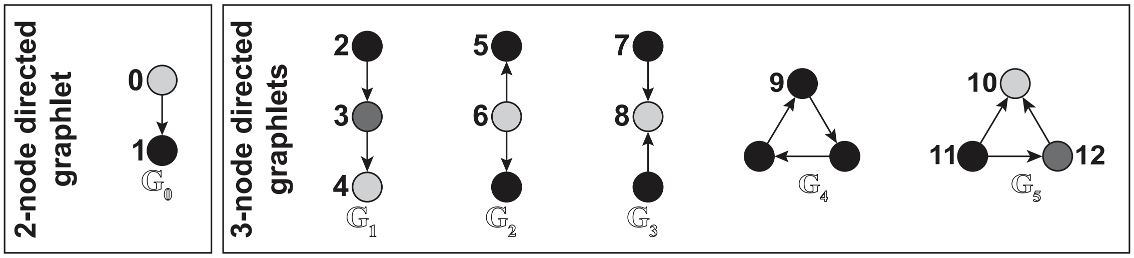

Fig A: The six directed graphlets (G_i) consisting of two to three nodes, and their respective orbits (i.e., the corresponding 13 numerically labelled node positions). Each unique shade in a single graphlet corresponds to a unique orbit in that graphlet.

Given a network, such as the one depicted in the first row of Fig B, we must first determine the number of times nodes occupy specific orbit positions. For DGCD-6, this amounts to counting the number of times nodes occupy the orbits of graphlets G_0 (orbits 0 and 1), G_2 (orbits 5 and 6), and G_3 (orbits 7 and 8). In tallying the number of times a node occupies orbital positions, the *directed graphlet degree vector* for a node is constructed. In Fig B, we provide the directed graphlet degree vector for node A.

Once directed graphlet degree vectors for each node in a network are determined (where a single directed graphlet degree vector is highlighted in red in Fig C), the *directed graphlet correlation matrix* can be assembled. In doing so, all possible Spearman's correlations between the number of times all nodes in a network occupy specific orbits are evaluated (see the highlighted green boxes in Fig C as an example of a single Spearman's correlation).

By computing the pairwise Euclidean distances between directed graphlet correlation matrices, we can obtain an estimate of the topological differences between networks in a given set. An example of a single pairwise Euclidean distance between two directed graphlet correlation matrices (i.e., two networks) is shown in Fig D.

Using all pairwise Euclidean distances between networks (i.e., all pairwise DGCD-6 between networks/graphlet correlation matrices), we can visualize their dissimilarity by projecting their distances using multidimensional scaling (MDS; see Fig E for an example). We performed MDS using the `MDS` function in the Scikit-learn library of Python [1].

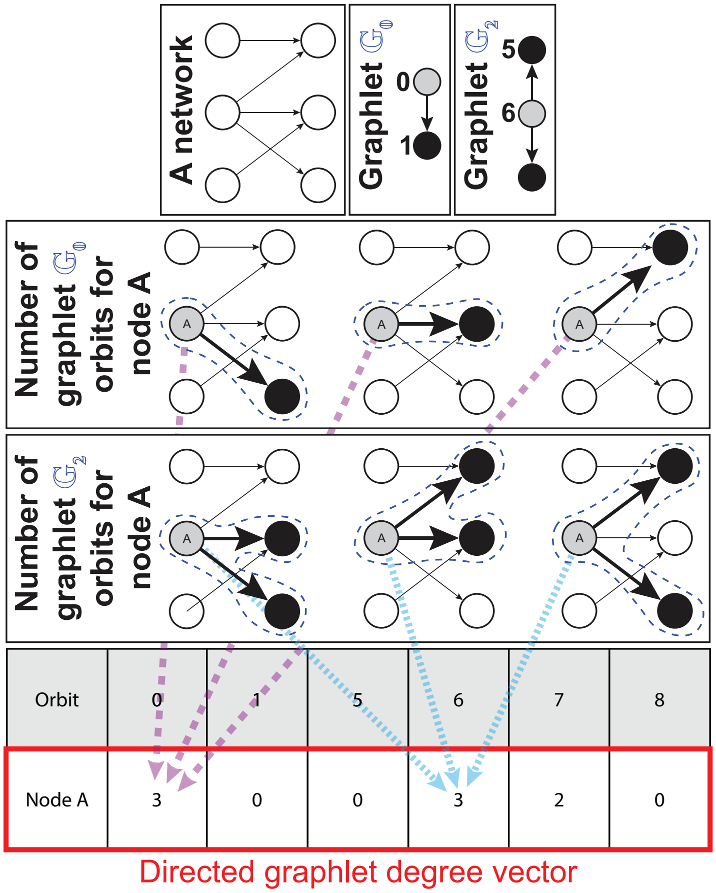

Fig B: Example calculation of a directed graphlet degree vector for a single node (node A) using the 6 orbits (i.e., 0, 1, 5, 6, 7, 8 of Fig A) that comprise the directed graphlet correlation distance 6 method.

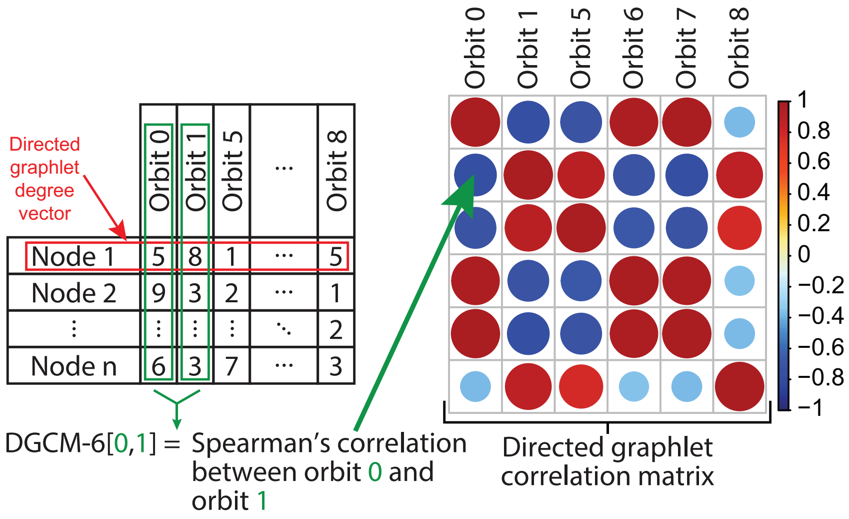

Fig C: Example calculation of a directed graphlet correlation matrix (DGCM-6) using 6 orbits that comprise the directed graphlet correlation distance 6 method. First, directed graphlet degree vectors for each node in a network are calculated (a single vector is highlighted in red). Next, Spearman's correlations are calculated between all pairs of orbits using the number of times each node occupies each orbit (an example of the vectors used in a single correlation is highlighted in green). The resulting correlations form entries within the directed DGCM-6.

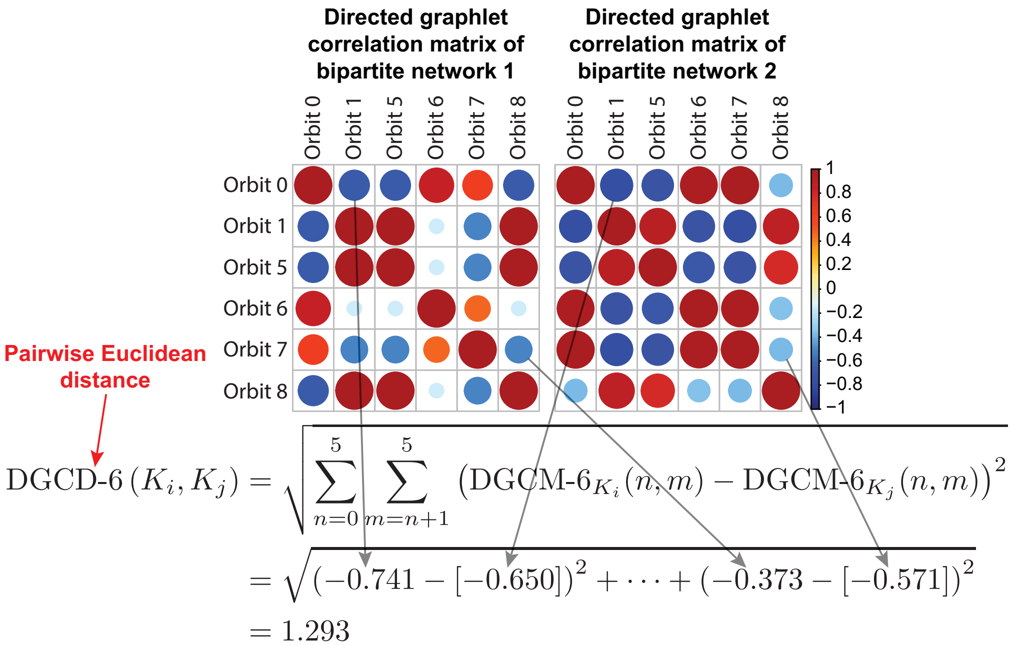

$$\mathrm{DGCD\text{-}6}\,(K_i, K_j) = \sqrt{\sum_{n=0}^{5} \sum_{m=n+1}^{5} \left(\mathrm{DGCM\text{-}6}_{K_i}(n,m) - \mathrm{DGCM\text{-}6}_{K_j}(n,m)\right)^2}$$
$$= \sqrt{(-0.741 - [-0.650])^2 + \cdots + (-0.373 - [-0.571])^2}$$
$$= 1.293$$

Fig D: The formula and an example calculation of the pairwise directed graphlet correlation distance 6 (DGCD-6) using the directed graphlet correlation matrices of two bipartite networks.

	Network 1	Network 2	⋯	Network n
Network 1	0.000	1.293	⋯	0.671
Network 2	1.293	0.000	⋯	1.039
⋮	⋮	⋮	⋱	⋮
Network n	0.671	1.039	⋯	0.000

Matrix of all pairwise DGCD-6 values between networks

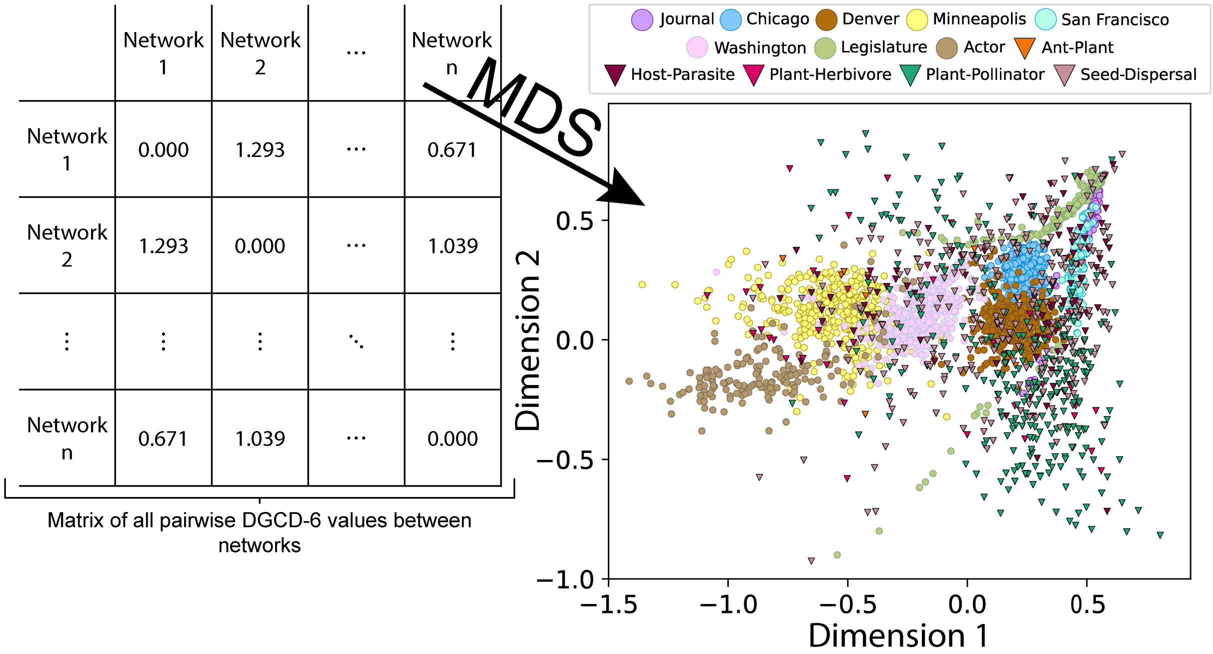

Fig E: An example of a 2-dimensional multidimensional scaling (MDS) projection of all pairwise DGCD-6s between a set of bipartite networks. This MDS projection is a subset of Fig G.

S1.2 Network general information

Table A: Additional bipartite network information. Subgrouping refers to a subgroup [i.e., networks classified as the same type of network from the same domain during network construction (e.g., the Chicago networks in the crime network domain)] that formed an obvious cluster within the MDS plot (Fig 4). See Table 2 for a list of network domains and their corresponding subgroups. All species interaction networks were classified into their appropriate subgroup even though they did not form subgroupings (e.g., Ant-plant).

Network domain	Subgrouping or subgroup	Mean connectance (Standard deviation)	Mean rows (Sd)	Mean columns (Sd)	Number of networks
Species interaction	Ant-plant	0.262 (0.082)	15.9 (9.632)	11.3 (13.039)	10
	Host-parasite	0.279 (0.13)	20.227 (30.054)	23.32 (20.057)	97
	Plant-herbivore	0.185 (0.107)	73.488 (171.286)	47.415 (122.832)	41
	Plant-pollinator	0.196 (0.096)	26.124 (52.037)	42.738 (74.494)	298
	Seed-diserpsal	0.303 (0.134)	23.599 (39.563)	17.032 (13.345)	277
	None[1]	0.249 (0.128)	26.91 (59.952)	30.115 (58.326)	723
Actor		0.005 (0.009)	7354.5 (8371.04)	1037.75 (1391.519)	172
Crime	Chicago	0.24 (0.018)	73.943 (1.694)	21.251 (1.595)	366
	Denver	0.06 (0.006)	55.85 (5.321)	47.858 (6.304)	366
	Minneapolis	0.162 (0.059)	27.735 (7.695)	10.12 (2.842)	366
	San Francisco	0.491 (0.038)	10.003 (0.052)	24.631 (1.95)	366
	Washington	0.303 (0.04)	29.929 (2.834)	6.779 (0.746)	366
Journal		0.04 (0.006)	389.349 (138.151)	623.138 (55.206)	109
Legislation		0.593 (0.139)	167.898 (161.857)	151.698 (198.062)	245
Microbiome		0.103 (0.025)	8625.236 (2607.786)	22.616 (7.693)	203
Sports	Baseball	0.002 (0.001)	23.972 (5.144)	930.563 (278.695)	71
	Basketball	0.012 (0.008)	16.618 (7.87)	246.794 (135.802)	68
	Hockey	0.004 (0.003)	21.818 (7.855)	697.364 (272.129)	55

[1] All species interaction networks without subgroups.

S1.3 DGCD-13 information

Table B: Median pairwise directed graphlet correlation distance 13 (DGCD-13) between bipartite networks from the same domain or subgrouping. Subgrouping refers to a subgroup [i.e., networks classified as the same type of network from the same domain during network construction (e.g., the Chicago networks in the crime network domain)] that formed an obvious cluster within the MDS plot (Fig 4). See Table 2 for a list of network domains and their corresponding subgroups. All species interaction networks were classified into their appropriate subgroup even though they did not form subgroupings (e.g., Ant-plant).

Network domain	Subgrouping or subgroup	Median pairwise DGCD-13	Number of networks
Species interaction	Ant-plant	0.744	10
	Host-parasite	1.011	97
	Plant-herbivore	1.260	41
	Plant-pollinator	0.802	298
	Seed-dispersal	1.032	277
	None[1]	1.042	723
Actor		0.359	172
Crime	Chicago	0.122	366
	Denver	0.167	366
	Minneapolis	0.431	366
	San Francisco	0.146	366
	Washington	0.277	366
Journal		0.123	109
Legislation		0.482	245
Microbiome		0.233	203
Sports	Baseball	0.197	71
	Basketball	0.399	68
	Hockey	0.254	55

[1] DGCD-13 between all species interaction networks.

Table C: Mean pairwise directed graphlet correlation distance 13 (DGCD-13) between bipartite species interaction networks from the same publication grouping. Bipartite networks sourced from publications that produced only a single network are termed "one network per publication".

Network domain	Bipartite type(s)/ subgroup	Publication grouping	Mean pairwise DGCD-13	Number of networks
Species interaction	Ant-plant, host-parasite, plant-herbivore, plant-pollinator, seed-dispersal	One network per publication	1.134	236
	Ant-plant	Passmore et al. (2012) [2]	0.622	8
	Host-parasite	Arai & Mudry (1983) [3]	0.387	2
		Kirjušina & Vismanis (2007) [4]	0.717	2
		Violante-González et al. (2007) [5]	0.160	2
		Hadfield et al. (2014) [6]	0.765	47
		Pilosof et al. (2013) [7]	0.214	6
	Host-parasite, plant-herbivore	Macfadyen et al. (2009) [8]	0.608	33
	Plant-herbivore	Henneman & Memmott (2001) [9]	0.411	2
		Pereira Martins et al. (2020) [10]	0.350	9
	Plant-pollinator	Arroyo et al. (1982) [11]	0.481	3
		Brosi et al. (2017) [12]	0.562	30
		Carstensen et al. (2018) [13]	0.478	14
		Dicks et al. (2002) [14]	0.260	2
		Dupont & Olesen (2009) [15]	0.254	2
		Gilarranz et al. (2015) [16]	0.283	12
		Kaiser-Bunbury et al. (2010) [17]	0.585	24
		Kaiser-Bunbury et al. (2014) [18]	0.605	44
		Lara-Romero et al. (2016) [19]	0.514	38
		Norfolk et al. (2018) [20]	0.516	2
		Maglianesi et al. (2014) [21]	0.475	3
		Magrach et al. (2018) [22]	0.298	12
		Medan et al. (2002) [23]	0.295	2
		Olesen et al. (2002) [24]	0.429	2
		Orford et al. (2016) [25]	0.564	37
		Primack (1983) [26]	0.361	3
		Sabatino et al. (2010) [27]	0.336	5
		Tinoco et al. (2017) [28]	0.737	3
		Trøjelsgaard et al. (2015) [29]	0.454	14
		Tur et al. (2013) [30]	0.191	2
	Seed-dispersal	Albrecht et al. (2015) [31]	0.525	16
		Carlo et al. (2003) [32]	0.284	4
		Chama et al. (2013) [33]	0.321	9
		Chaves (2018) [34]	0.960	2
		Correa et al. (2016) [35]	0.554	2
		Correia et al. (2017) [36]	0.674	2
		Cruz et al. (2013) [37]	0.633	3
		Ribeiro da Silva et al. (2015) [38]	0.747	3
		Dehling et al. (2014) [39]	0.527	8
		Farwig et al. (2017) [40]	0.190	2
		Galetti & Pizo (1996) [41]	1.230	2
		García et al. (2014) [42]	0.561	6
		Innis (1989) [43]	0.575	2
		Malmborg & Willson (1988) [44]	0.623	3
		Menke et al. (2012) [45]	0.220	4
		Passos et al. (2003) [46]	0.673	2
		Peredo et al. (2013) [47]	1.084	2
		Plein et al. (2013) [48]	0.358	5
		Purificação et al. (2014) [49]	0.627	3
		Quitián et al. (2019) [50]	0.473	4
		Ramos-Robles et al. (2016) [51]	0.634	7
		Ruggera et al. (2016) [52]	0.536	10
		Saavedra et al. (2014) [53]	0.556	2
		Del Valle (2014) [54]	0.238	2
		Snow & Snow (1988) [55]	0.781	4
		Gomes (2008) [56]	0.357	2
		Velho et al. (2012) [57]	0.272	2
		Vizentin-Bugoni et al. (2019) [58]	0.687	7
		Williams & Karl (1996) [59]	0.767	2

S1.3.1 DGCD-13 as a function of the number of species interaction networks sourced from each publication (for publications that provide more than a single network)

It was possible that the mean pairwise DGCD-13 for publications which provided more than a single species interaction network were influenced by the number of networks sourced from each publication. For example, it could have been the case that publications that each only provided two species interaction networks had lower mean pairwise DGCD-13 between their own networks than publications that each provided ten species interaction networks. However, we found no strong relationships between the number of networks a publication provided and the mean pairwise DGCD-13. Specifically, when dividing publications (which provided more than a single network) into quartiles based on the number of networks each provided, there were no large differences in mean pairwise DGCD-13 between quartiles (Fig F). Thus, the mean pairwise DGCD-13 for species interaction networks sharing a publication source was not strongly influenced by the number of networks each publication provided.

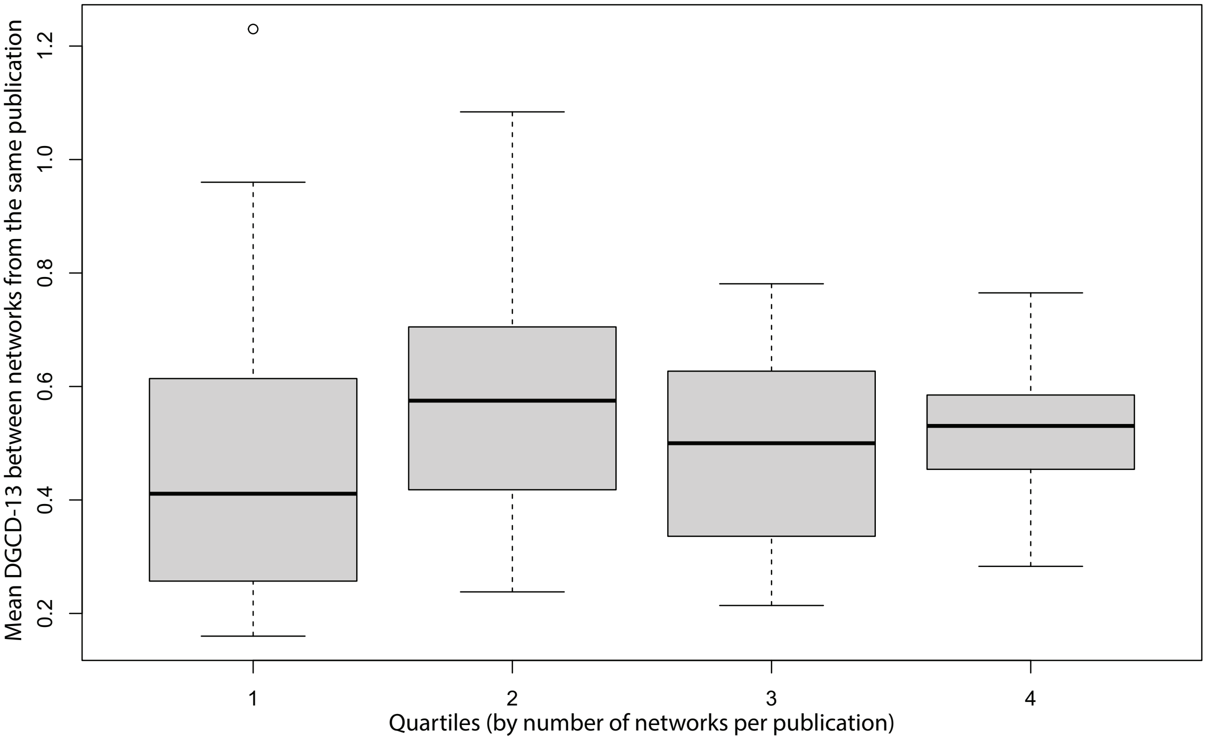

Fig F: The relationship between the mean pairwise DGCD-13 of species interaction networks sourced from the same publication ($n = 487$) and the number of networks a publication provided (represented by quartiles). Note that each publication included in the analyses provided at least two networks. See Table C for a list of publications and the number of networks each provided. The data and code needed to generate this Figure can be found in `www.osf.io/my9tv`.

S1.3.2 DGCD-13 as a function of the variability in species interaction network size

It could have been the case that the larger variability in species interaction network size (i.e., number of nodes) caused its larger mean pairwise DGCD-13, as compared to other non-ecological networks which generally had less variability in network size. To test whether this was true, we divided species interaction networks into quartile groups (by network size) to reduce variability in size across a group. We then evaluated the mean pairwise DGCD-13 between the smallest networks (i.e., those within the first quartile) and the largest networks (i.e., those within the fourth quartile) (Table D). We removed any networks sourced from the same publication to eliminate the effect of publication in this analysis. If multiple networks were sourced from the same publication within a quartile, we only kept a single network from that publication. A single network was chosen using a simple criterion: first, we kept the network with the largest size. If more than one network from the same publication had the same size, we then chose the network with the highest connectance value. If again, more than one network had the same connectance, we chose the network with the greatest number of rows in its adjacency matrix.

Altogether, we found that the standard deviation in network size for the first quartile (representing the smallest networks in our analyses) was considerably smaller in relation to its mean network size as compared to the fourth quartile (representing the largest networks in our analyses). Specifically, the first quartile had a standard deviation in network size of 4.259 and mean network size of 18.544 while the fourth quartile had a standard deviation in network size of 217.844 and mean network size of 170.652 (Table D). However, the first quartile had a larger mean pairwise DGCD-13 than the fourth quartile (1.088 vs. 0.982, respectively) which would not have been expected if the variability in network size influenced mean pairwise DGCD-13. Hence, it does not appear that the variability of network size strongly affected the mean pairwise DGCD-13 in species interaction networks.

Table D: Pairwise directed graphlet correlation distance 13 (DGCD-13) between species interaction networks using the smallest and largest networks classified into quartiles (whereby size was determined by number of nodes).

Network domain	Size quartile	Mean network size (Std. dev.)	Mean pairwise DGCD-13 (Std. dev.)	Number of networks[1]
Species interaction	1	18.544 (4.259)	1.088 (0.464)	90
	4	170.652 (217.844)	0.982 (0.507)	112

[1] All networks included in each quartile were from different publications.

S1.4 DGCD-6 information

Here, we reanalyzed the same bipartite networks as those in the main manuscript but instead with the directed graphlet correlation distance that used only the six orbits (termed DGCD-6) from graphlets G_0, G_2, and G_3—specifically, orbits 0, 1, 5, 6, 7, and 8 (Fig A). Generally, the same results were obtained as in the main manuscript: species interaction networks were still the most topologically heterogeneous networks (mean pairwise DGCD-6 of 0.620)—about 76% more heterogeneous than the domain with the second most heterogeneity (i.e., Actor networks with mean pairwise DGCD-6 of 0.352) (Table E and Fig G). When instead using median pairwise DGCD-6 (Table F), these results still held, and so the large mean pairwise DGCD-6 found between species interaction networks was not primarily driven by outliers. Moreover, exclusively within the species interaction domain, networks from the same publication were much more topologically similar (by a factor of about 2) than networks that were each a product of their own publication (0.331 and 0.634 mean pairwise DGCD-6, respectively, Table G).

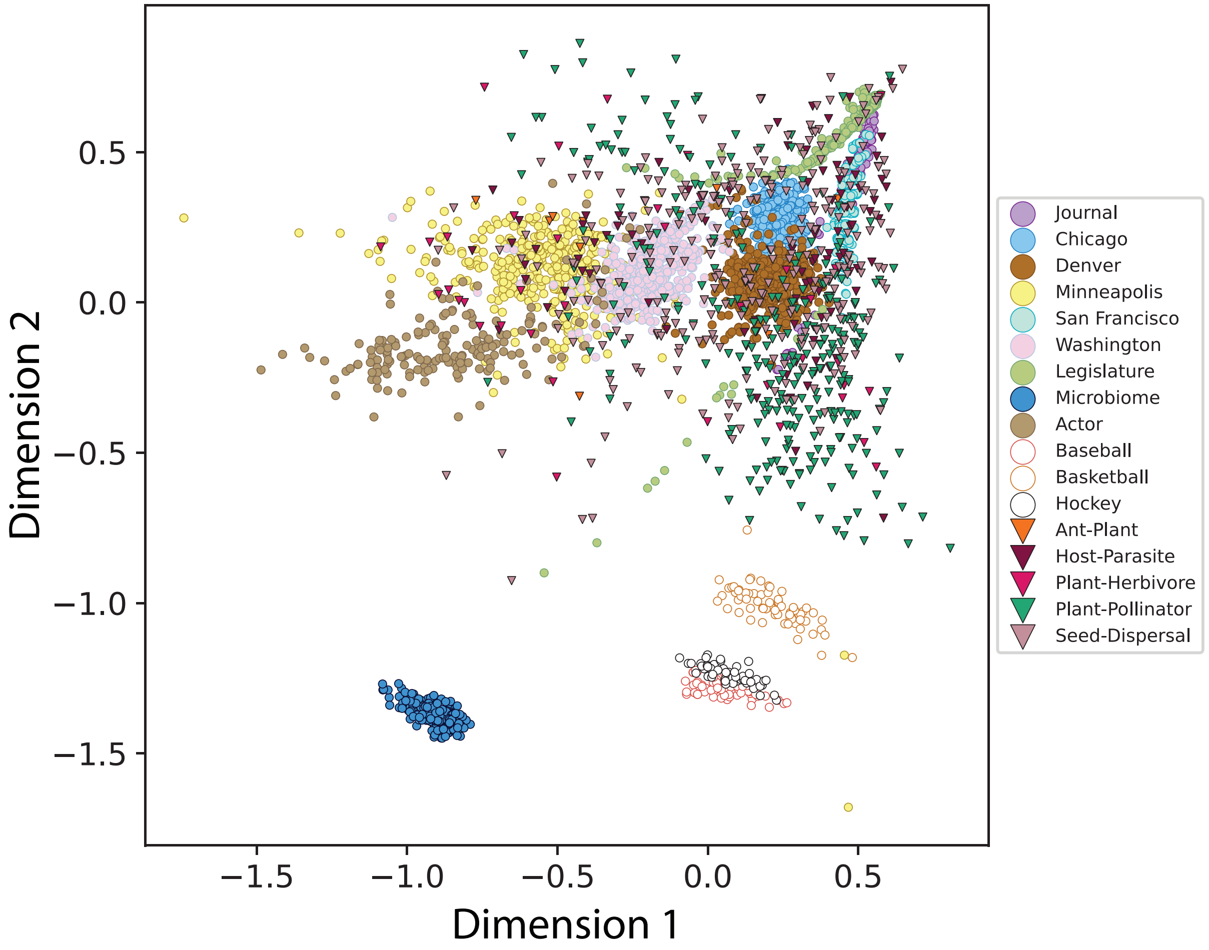

Fig G: Multidimensional scaling of the pairwise directed graphlet correlation distance 6 (DGCD-6) between all bipartite networks ($n = 3476$). Each point in plot is a single network. The data and code needed to generate this Figure can be found in `www.osf.io/my9tv`.

Table E: Mean pairwise directed graphlet correlation distance 6 (DGCD-6) between bipartite networks from the same domain or subgrouping. Subgrouping refers to a subgroup [i.e., networks classified as the same type of network from the same domain during network construction (e.g., the Chicago networks in the crime network domain)] that formed an obvious cluster within the MDS plot (Fig G). See Table 2 for a list of network domains and their corresponding subgroups. All species interaction networks were classified into their appropriate subgroup even though they did not form subgroupings (e.g., Ant-plant).

Network domain	Subgrouping or subgroup	Mean pairwise DGCD-6	Number of networks
Species interaction	Ant-plant	0.543	10
	Host-parasite	0.586	97
	Plant-herbivore	0.774	41
	Plant-pollinator	0.526	298
	Seed-dispersal	0.567	277
	None[1]	0.620	723
Actor		0.352	172
Crime	Chicago	0.105	366
	Denver	0.150	366
	Minneapolis	0.334	366
	San Francisco	0.127	366
	Washington	0.217	366
Journal		0.214	109
Legislation		0.300	245
Microbiome		0.115	203
Sports	Baseball	0.130	71
	Basketball	0.189	68
	Hockey	0.134	55

[1] DGCD-6 between all species interaction networks.

Table F: Median pairwise directed graphlet correlation distance 6 (DGCD-6) between bipartite networks from the same domain or subgrouping. Subgrouping refers to a subgroup [i.e., networks classified as the same type of network from the same domain during network construction (e.g., the Chicago networks in the crime network domain)] that formed an obvious cluster within the MDS plot (Fig G). See Table 2 for a list of network domains and their corresponding subgroups. All species interaction networks were classified into their appropriate subgroup even though they did not form subgroupings (e.g., Ant-plant).

Network domain	Subgrouping or subgroup	Median pairwise DGCD-6	Number of networks
Species interaction	Ant-plant	0.507	10
	Host-parasite	0.555	97
	Plant-herbivore	0.784	41
	Plant-pollinator	0.487	298
	Seed-dispersal	0.531	277
	None[1]	0.589	723
Actor		0.306	172
Crime	Chicago	0.096	366
	Denver	0.138	366
	Minneapolis	0.295	366
	San Francisco	0.108	366
	Washington	0.198	366
Journal		0.113	109
Legislation		0.194	245
Microbiome		0.097	203
Sports	Baseball	0.111	71
	Basketball	0.173	68
	Hockey	0.125	55

[1] DGCD-6 between all species interaction networks.

Table G: Mean pairwise directed graphlet correlation distance 6 (DGCD-6) of bipartite species interaction networks from the same publication grouping. Bipartite networks sourced from the same publication are termed "multiple networks per publication" and bipartite networks sourced from publications that each produced only a single network are termed "one network per publication". See Table H for a list of publications that provided more than one network and each publication's mean pairwise DGCD-6.

Publication grouping	Mean pairwise DGCD-6	Number of networks	Number of publications
One network per publication	0.634	236	236
Multiple networks per publication	0.331[1]	487	58

[1] Calculated by taking the mean of the average pairwise DGCD-6s between networks from the same publication, weighted by the number of networks produced by each publication.

Table H: Mean pairwise directed graphlet correlation distance 6 (DGCD-6) between bipartite species interaction networks from the same publication grouping. Bipartite networks sourced from publications that produced only a single network are termed "one network per publication".

Network domain	Bipartite type(s)/ subgroup	Publication grouping	Mean pairwise DGCD-6	Number of networks
Species interaction	Ant-plant, host-parasite, plant-herbivore, plant-pollinator, seed-dispersal	One network per publication	0.634	236
	Ant-plant	Passmore et al. (2012) [2]	0.457	8
	Host-parasite	Arai & Mudry (1983) [3]	0.322	2
		Kirjušina & Vismanis (2007) [4]	0.330	2
		Violante-González et al. (2007) [5]	0.047	2
		Hadfield et al. (2014) [6]	0.369	47
		Pilosof et al. (2013) [7]	0.144	6
	Host-parasite, plant-herbivore	Macfadyen et al. (2009) [8]	0.420	33
	Plant-herbivore	Henneman & Memmott (2001) [9]	0.291	2
		Pereira Martins et al. (2020) [10]	0.233	9
	Plant-pollinator	Arroyo et al. (1982) [11]	0.312	3
		Brosi et al. (2017) [12]	0.335	30
		Carstensen et al. (2018) [13]	0.328	14
		Dicks et al. (2002) [14]	0.199	2
		Dupont & Olesen (2009) [15]	0.152	2
		Gilarranz et al. (2015) [16]	0.209	12
		Kaiser-Bunbury et al. (2010) [17]	0.337	24
		Kaiser-Bunbury et al. (2014) [18]	0.354	44
		Lara-Romero et al. (2016) [19]	0.350	38
		Norfolk et al. (2018) [20]	0.227	2
		Maglianesi et al. (2014) [21]	0.297	3
		Magrach et al. (2018) [22]	0.174	12
		Medan et al. (2002) [23]	0.252	2
		Olesen et al. (2002) [24]	0.288	2
		Orford et al. (2016) [25]	0.360	37
		Primack (1983) [26]	0.224	3
		Sabatino et al. (2010) [27]	0.216	5
		Tinoco et al. (2017) [28]	0.470	3
		Trøjelsgaard et al. (2015) [29]	0.286	14
		Tur et al. (2013) [30]	0.185	2
	Seed-dispersal	Albrecht et al. (2015) [31]	0.315	16
		Carlo et al. (2003) [32]	0.195	4
		Chama et al. (2013) [33]	0.219	9
		Chaves (2018) [34]	0.667	2
		Correa et al. (2016) [35]	0.377	2
		Correia et al. (2017) [36]	0.238	2
		Cruz et al. (2013) [37]	0.434	3
		Ribeiro da Silva et al. (2015) [38]	0.498	3
		Dehling et al. (2014) [39]	0.231	8
		Farwig et al. (2017) [40]	0.144	2
		Galetti & Pizo (1996) [41]	0.777	2
		García et al. (2014) [42]	0.370	6
		Innis (1989) [43]	0.476	2
		Malmborg & Willson (1988) [44]	0.255	3
		Menke et al. (2012) [45]	0.143	4
		Passos et al. (2003) [46]	0.279	2
		Peredo et al. (2013) [47]	0.757	2
		Plein et al. (2013) [48]	0.237	5
		Purificação et al. (2014) [49]	0.346	3
		Quitián et al. (2019) [50]	0.241	4
		Ramos-Robles et al. (2016) [51]	0.372	7
		Ruggera et al. (2016) [52]	0.329	10
		Saavedra et al. (2014) [53]	0.391	2
		Del Valle (2014) [54]	0.152	2
		Snow & Snow (1988) [55]	0.386	4
		Gomes (2008) [56]	0.233	2
		Velho et al. (2012) [57]	0.210	2
		Vizentin-Bugoni et al. (2019) [58]	0.421	7
		Williams & Karl (1996) [59]	0.631	2

References

[1] Pedregosa F, Varoquaux G, Gramfort A, Michel V, Thirion B, Grisel O, et al. Scikit-learn: Machine Learning in Python. Journal of Machine Learning Research. 2011;12:2825–2830.

[2] Passmore HA, Bruna EM, Heredia SM, Vasconcelos HL. Resilient networks of ant-plant mutualists in Amazonian forest fragments. PLOS ONE. 2012;7(8):e40803.

[3] Arai HP, Mudry DR. Protozoan and metazoan parasites of fishes from the headwaters of the Parsnip and McGregor Rivers, British Columbia: A study of possible parasite transfaunations. Canadian Journal of Fisheries and Aquatic Sciences. 1983;40(10):1676–1684.

[4] Kirjušina M, Vismanis K. Checklist of the parasites of fishes of Latvia. FAO Fisheries Technical Paper. No. 369/3; 2007.

[5] Violante-González J, Aguirre-Macedo ML, Mendoza-Franco EF. A checklist of metazoan parasites of fish from Tres Palos Lagoon, Guerrero, Mexico. Parasitology Research. 2007;102(1):151–161.

[6] Hadfield JD, Krasnov BR, Poulin R, Nakagawa S. A tale of two phylogenies: Comparative analyses of ecological interactions. The American Naturalist. 2014;183(2):174–187.

[7] Pilosof S, Fortuna MA, Vinarski MV, Korallo-Vinarskaya NP, Krasnov BR. Temporal dynamics of direct reciprocal and indirect effects in a host–parasite network. Journal of Animal Ecology. 2013;82(5):987–996.

[8] Macfadyen S, Gibson R, Polaszek A, Morris RJ, Craze PG, Planqué R, et al. Do differences in food web structure between organic and conventional farms affect the ecosystem service of pest control? Ecology Letters. 2009;12(3):229–238.

[9] Henneman ML, Memmott J. Infiltration of a Hawaiian community by introduced biological control agents. Science. 2001;293(5533):1314–1316.

[10] Pereira Martins L, Matos Medina A, Lewinsohn TM, Almeida-Neto M. The effect of species composition dissimilarity on plant–herbivore network structure is not consistent over time. Biotropica. 2020;52(4):664–674.

[11] Arroyo MTK, Primack R, Armesto J. Community studies in pollination ecology in the high temperate Andes of central Chile. I. Pollination mechanisms and altitudinal variation. American Journal of Botany. 1982;69(1):82–97.

[12] Brosi BJ, Niezgoda K, Briggs HM. Experimental species removals impact the architecture of pollination networks. Biology Letters. 2017;13(6):20170243.

[13] Carstensen DW, Trøjelsgaard K, Ollerton J, Morellato LPC. Local and regional specialization in plant–pollinator networks. Oikos. 2018;127(4):531–537.

[14] Dicks LV, Corbet SA, Pywell RF. Compartmentalization in plant-insect flower visitor webs. Journal of Animal Ecology. 2002;71(1):32–43.

[15] Dupont YL, Olesen JM. Ecological modules and roles of species in heathland plant-insect flower visitor networks. Journal of Animal Ecology. 2009;78(2):346–353.

[16] Gilarranz LJ, Sabatino M, Aizen MA, Bascompte J. Hot spots of mutualistic networks. Journal of Animal Ecology. 2015;84(2):407–413.

[17] Kaiser-Bunbury CN, Muff S, Memmott J, Müller CB, Caflisch A. The robustness of pollination networks to the loss of species and interactions: A quantitative approach incorporating pollinator behaviour. Ecology Letters. 2010;13(4):442–452.

[18] Kaiser-Bunbury CN, Vázquez DP, Stang M, Ghazoul J. Determinants of the microstructure of plant–pollinator networks. Ecology. 2014;95(12):3314–3324.

[19] Lara-Romero C, García C, Morente-López J, Iriondo JM. Direct and indirect effects of shrub encroachment on alpine grasslands mediated by plant–flower visitor interactions. Functional Ecology. 2016;30(9):1521–1530.

[20] Norfolk O, Gilbert F, Eichhorn MP. Alien honeybees increase pollination risks for range-restricted plants. Diversity and Distributions. 2018;24(5):705–713.

[21] Maglianesi MA, Blöthgen N, Böhning-Gaese K, Schleuning M. Morphological traits determine specialization and resource use in plant–hummingbird networks in the neotropics. Ecology. 2014;95(12):3325–3334.

[22] Magrach A, Holzschuh A, Bartomeus I, Riedinger V, Roberts SPM, Rundlöf M, et al. Plant-pollinator networks in semi-natural grasslands are resistant to the loss of pollinators during blooming of mass-flowering crops. Ecography. 2018;41(1):62–74.

[23] Medan D, Montaldo NH, Devoto M, Mantese A, Vasellati V, Roitman GG, et al. Plant-pollinator relationships at two altitudes in the Andes of Mendoza, Argentina. Arctic, Antarctic, and Alpine Research. 2002;34(3):233–241.

[24] Olesen JM, Eskildsen LI, Venkatasamy S. Invasion of pollination networks on oceanic islands: Importance of invader complexes and endemic super generalists. Diversity and Distributions. 2002;8(3):181–192.

[25] Orford KA, Murray PJ, Vaughan IP, Memmott J. Modest enhancements to conventional grassland diversity improve the provision of pollination services. Journal of Applied Ecology. 2016;53(3):906–915.

[26] Primack RB. Insect pollination in the New Zealand mountain flora. New Zealand Journal of Botany. 1983;21(3):317–333.

[27] Sabatino M, Maceira N, Aizen MA. Direct effects of habitat area on interaction diversity in pollination webs. Ecological Applications. 2010;20(6):1491–1497.

[28] Tinoco BA, Graham CH, Aguilar JM, Schleuning M. Effects of hummingbird morphology on specialization in pollination networks vary with resource availability. Oikos. 2017;126(1):52–60.

[29] Trøjelsgaard K, Jordano P, Carstensen DW, Olesen JM. Geographical variation in mutualistic networks: Similarity, turnover and partner fidelity. Proceedings of the Royal Society B: Biological Sciences. 2015;282(1802):20142925.

[30] Tur C, Vigalondo B, Trøjelsgaard K, Olesen JM, Traveset A. Downscaling pollen-transport networks to the level of individuals. Journal of Animal Ecology. 2013;83(1):306–317.

[31] Albrecht J, Bohle V, Berens DG, Jaroszewicz B, Selva N, Farwig N. Variation in neighbourhood context shapes frugivore-mediated facilitation and competition among co-dispersed plant species. Journal of Ecology. 2015;103(2):526–536.

[32] Carlo TA, Collazo JA, Groom MJ. Avian fruit preferences across a Puerto Rican forested landscape: Pattern consistency and implications for seed removal. Oecologia. 2003;134(1):119–131.

[33] Chama L, Berens DG, Downs CT, Farwig N. Habitat Characteristics of Forest Fragments Determine Specialisation of Plant-Frugivore Networks in a Mosaic Forest Landscape. PLOS ONE. 2013;8(1):1–6.

[34] Chaves PAP. Response of avian and mammal seed dispersal networks, to human induced forest edges in a sub-humid forest [Thesis]. Universidade de Lisboa; 2018.

[35] Correa SB, Arujo JK, Penha J, Nunes da Cunha C, Bobier KE, Anderson JT. Stability and generalization in seed dispersal networks: A case study of frugivorous fish in Neotropical wetlands. Proceedings of the Royal Society B: Biological Sciences. 2016;283(1837):20161267.

[36] Correia M, Timóteo S, Rodríguez-Echeverría S, Mazars-Simon A, Heleno RH. Refaunation and the reinstatement of the seed-dispersal function in Gorongosa National Park. Conservation Biology. 2017;31(1):76–85.

[37] Cruz JC, Ramos JA, Da Silva LP, Tenreiro PQ, Heleno RH. Seed dispersal networks in an urban novel ecosystem. European Journal of Forest Research. 2013;132(5):887–897.

[38] Ribeiro da Silva F, Montoya D, Furtado R, Memmott J, Pizo MA, Rodrigues RR. The restoration of tropical seed dispersal networks. Restoration Ecology. 2015;23(6):852–860.

[39] Dehling DM, Fritz SA, Töpfer T, Päckert M, Estler P, Böhning-Gaese K, et al. Functional and phylogenetic diversity and assemblage structure of frugivorous birds along an elevational gradient in the tropical Andes. Ecography. 2014;37(11):1047–1055.

[40] Farwig N, Schabo DG, Albrecht J. Trait-associated loss of frugivores in fragmented forest does not affect seed removal rates. Journal of Ecology. 2017;105(1):20–28.

[41] Galetti M, Pizo MA. Fruit eating by birds in a forest fragment in southeastern Brazil. Revista Brasileira de Ornitologia. 1996;4(2):71–79.

[42] García D, Martínez D, Stouffer DB, Tylianakis JM. Exotic birds increase generalization and compensate for native bird decline in plant–frugivore assemblages. Journal of Animal Ecology. 2014;83(6):1441–1450.

[43] Innis GJ. Feeding ecology of fruit pigeons in subtropical rainforests of south-eastern Queensland. Wildlife Research. 1989;16(4):365–394.

[44] Malmborg PK, Willson MF. Foraging Ecology of Avian Frugivores and Some Consequences for Seed Dispersal in an Illinois Woodlot. The Condor. 1988;90(1):173–186.

[45] Menke S, Böhning-Gaese K, Schleuning M. Plant–frugivore networks are less specialized and more robust at forest–farmland edges than in the interior of a tropical forest. Oikos. 2012;121(10):1553–1566.

[46] Passos FC, Silva WR, Pedro WA, Bonin MR. Frugivoria em morcegos (Mammalia, Chiroptera) no Parque Estadual intervales, sudeste do Brasil. Revista Brasileira de Zoologia. 2003;20(3):511–517.

[47] Peredo A, Martínez D, Rodríguez-Pérez J, García D. Mammalian seed dispersal in Cantabrian woodland pastures: Network structure and response to forest loss. Basic and Applied Ecology. 2013;14(5):378–386.

[48] Plein M, Längsfeld L, Neuschulz EL, Schultheiß C, Ingmann L, Töpfer T, et al. Constant properties of plant–frugivore networks despite fluctuations in fruit and bird communities in space and time. Ecology. 2013;94(6):1296–1306.

[49] Purificação KN, Pascotto MC, Pedroni F, Pereira JMN, Lima NA. Interactions between frugivorous birds and plants in savanna and forest formations of the Cerrado. Biota Neotropica. 2014;14(4):e20140068.

[50] Quitián M, Santillán V, Bender IMA, Espinosa CI, Homeier J, Böhning-Gaese K, et al. Functional responses of avian frugivores to variation in fruit resources between natural and fragmented forests. Functional Ecology. 2019;33(3):399–410.

[51] Ramos-Robles M, Andresen E, Díaz-Castelazo C. Temporal changes in the structure of a plant-frugivore network are influenced by bird migration and fruit availability. PeerJ. 2016;4:e2048.

[52] Ruggera RA, Blendinger PG, Gomez MD, Marshak C. Linking structure and functionality in mutualistic networks: Do core frugivores disperse more seeds than peripheral species? Oikos. 2016;125(4):541–555.

[53] Saavedra F, Hensen I, Beck SG, Böhning-Gaese K, Lippok D, Töpfer T, et al. Functional importance of avian seed dispersers changes in response to human-induced forest edges in tropical seed-dispersal networks. Oecologia. 2014;176(3):837–848.

[54] Del Valle MTS. Dispersión de semillas por quirópteros en hualares de la zona Uxpanapa, Veracruz, México [Thesis]. Universidad Veracruzana; 2014.

[55] Snow B, Snow D. Birds and berries. London, U.K.: A&C Black; 1988.

[56] Gomes ALS. Interação mutualística entre aves frugívoras de sub-bosque e plantas no Parque Ecológico de Gunma, Santa Bárbara do Pará [Thesis]. Universidade Federal do Pará; 2008.

[57] Velho N, Ratnam J, Srinivasan U, Sankaran M. Shifts in community structure of tropical trees and avian frugivores in forests recovering from past logging. Biological Conservation. 2012;153:32–40.

[58] Vizentin-Bugoni J, Tarwater CE, T FJ, Drake DR, Gleditsch JM, Hruska AM, et al. Structure, spatial dynamics, and stability of novel seed dispersal mutualistic networks in Hawai'i. Science. 2019;364(6435):78–82.

[59] Williams PA, Karl BJ. Fleshy fruits of indigenous and adventive plants in the diet of birds in forest remnants, Nelson, New Zealand. New Zealand Journal of Ecology. 1996; p. 127–145.
