# Supplementary material for: Shortcomings of reusing species interaction networks created by different sets of researchers
Source: PLoS Biol. 2023 Apr 3;21(4):e3002068. doi: 10.1371/journal.pbio.3002068 (PMC10101633; doi:10.1371/journal.pbio.3002068)
Supplement: Attachment — Submitted filename: Response_to_reviews.pdf [file pbio.3002068.s002.pdf]

Editor-in-Chief
PLOS Biology
August 17, 2022

Dear Dr. Nonia Pariente,

We look forward to receiving reviews back on our paper. We are including a document entitled "Response to Reviewers" since it is required for submission to *PLOS Biology*.

Please note that we were advised to change our manuscript from a *research article* to a *meta-research article*, but we are currently unable to do so on the website (there is no option for *meta-research article*).

Thanks,

Chris Brimacombe
