# Supplementary material for: Shortcomings of reusing species interaction networks created by different sets of researchers
Source: PLoS Biol. 2023 Apr 3;21(4):e3002068. doi: 10.1371/journal.pbio.3002068 (PMC10101633; doi:10.1371/journal.pbio.3002068)
Supplement: Attachment — Submitted filename: Response_to_reviewers_Jan_15.docx [file pbio.3002068.s003.docx]

**[Reviewer #1, Dr. Miguel A. Fortuna]:** I have read the manuscript on the meta-study performed to highlight the importance of considering the publication source when reusing and analyzing species interaction networks. I think this is the main message of this manuscript. It is already known that the species interaction networks available in databases, like the Web of Life database, are highly heterogenous and that a careful consideration should be taken when comparing network topology across networks. The authors quantified that heterogeneity with a novel metric and compare the similarity of species interaction networks with the similarity of non-biological networks that were built using a much more standardized approach. That is novel and interesting.

**[Response]:** We thank Dr. Miguel A. Fortuna for their helpful comments. We have tracked all additional text in the manuscript by including them as orange. All unchanged text in the manuscript is written in black, and all of our responses (in this file) are written in blue.

We agree that heterogeneity in online databases for ecological networks is an important topic and we are glad that you appreciated our approach to quantifying the topological heterogeneity. We acknowledge that the issue of heterogeneity in networks has been previously discussed (e.g., Paine 1988, Polis 1991) but also we recognize that this issue has largely been relegated to the backburner as often heterogeneities are ignored in network comparisons.

In reading your review, we realized that we omitted important citations that had also emphasized the need for caution when reusing networks and so we have now added these to the Introduction of our manuscript, starting on LN 19: “A current crux of reusing available species interaction networks created by different sets of researchers, however, is the unwanted topological differences that can exist due to the lack of consistency in the way ecological systems are translated into networks by different sets of researchers (Dormann et al., 2009; Ings et al., 2009; Gibson et al., 2011; Doré et al., 2021; Quintero et al., 2022; Salim et al., 2022; Mestre et al., 2022; Vázquez et al., 2022) (Figure 1). While some criticisms related to this issue had been raised in the 1980s/90s, e.g., Paine (1988); Polis (1991), with the increasing availability to the internet and growth in computational power, a renewed interest in networks was sparked, and many of these concerns were overlooked (Pringle and Hutchinson, 2020).”

**[Reviewer #1]:** In general, I like the paper and I think it meets the requirements for its publication. However, some methodological questions on the Directed Graphlet Correlation Distance (DGCD) should be clarified. Specifically, a new figure illustrating how those graphlets are embedded within a bipartite network (replacing current Figure 1). I also miss a discussion on to what extent high values of DGCD might or might not invalidate the existence of specific network patterns, like nestedness and modularity, that have been found in many species interaction networks.

**[Response]:** We are glad that you find the manuscript interesting! Given the number of species interaction network studies currently ongoing, especially those that reuse networks created by different sets of researchers, we thought it pertinent to highlight some of their pitfalls.

We have now amended Figure 3 (which we believe you meant instead of Figure 1), to show an example of how directed graphlets are embedded within bipartite networks. In doing so, we also became aware of the difficulty in conceptualizing the DGCD method from only in-text descriptions. As such, we have now introduced a new section in the S1 Appendix (Section S1.1) that describes in detail the calculations involved when applying this method. More details are provided in the comments below.

Finally, we recognize that our findings to not necessarily overturn all studies that have previously found relationships within reused networks created by different sets of researchers (as per your comment about nestedness, modularity, ect.). We have now directly addressed this in our Discussion as a new paragraph starting on LN 387: “Although we generally failed to find pervasive and coherent topology within species interaction networks created by different sets of researchers, we highlight that our results do not necessarily invalidate patterns others have found [e.g., high nestedness in plant-animal networks (Bascompte and Jordano, 2007)]. Instead, these patterns are perhaps true under strict conditions, such as controlling for the unwanted differences in topology between studies when reusing their networks.”

**[Reviewer #1]:** I think the authors have done a great job in this meta-study. I like the motivation of this manuscript, the novel approach they have followed to compare network topology, and the extent of the dataset they have used. Yet, I miss an explicit interpretation in the discussion section on how the global metric applied (DGCD) might or might not invalidate the existence of specific network patterns that have been widely detected across species interaction networks (e.g., nestedness and modularity). Some methodological questions should also be clarified. Below, I have listed some minor comments that I think will improve the reader's understanding.

**[Response]:** We address each comment below in detail (which covers each topic listed here).

**[Reviewer #1]:** I think current figure 1 does not clearly depict the idea underlying the directed graphlet correlation distance. Perhaps, a new one representing a couple of graphlets embedded within a small bipartite network would be much more informative.

**[Response]:** We agree, and we have now amended Figure 3 (which we believe you meant instead of Figure 1) to show an example of how graphlets are embedded within a bipartite network.

**[Reviewer #1]:** I would appreciate a clarification on the distinction between graphlets and network motifs.

**[Response]:** This is a fair point, and one for which we had addressed, but removed prior to submission. We have added it back to the manuscript as an additional paragraph to the Methods section that clarifies the differences between graphlets and motifs. Starting on LN 135: “In ecology, the most adopted subgraph technique is based on motifs. Generally, for a graph *G* composed of a set of nodes *V* and a set of links *L*, denoted as *G*(*V*, *L*); a motif of *G* is a subgraph *G’*(*V’*, *L’*) with a subset of nodes *V’* from *V* where any edges linking the nodes of *V’* found in *V* are contained in *L’*. Motif interpretation is highly dependent on the choice of a network null model since motifs are defined as small partial subgraphs that are over-represented in the real network compared to a chosen network null model (Pržulj et al., 2010; Yaveroglu et al., 2014). As a consequence, motifs have been criticized for possibly relying on ill-posed null models as a basis for significance testing (Artzy-Randrup et al., 2004).” In addition, we have appended extra statements to indicate that DGCD does not rely on null models for network comparison, starting on LN 144: “To overcome the null model limitation of motifs, we instead adopted the subgraph technique of directed graphlet correlation distance (DGCD) (Sarajlić et al., 2016) to characterize the topological differences between networks. Generally, DGCD evaluates network pairwise dissimilarity without relying on a network null model, and does so by quantifying differences in the associations between the appearance of directed graphlets (Figure 3) within a given network to those of another network.” Finally, we formally define graphlets, starting on LN 150: “Formally, graphlets are the induced subgraphs *G’*(*V’*, *L’*), consisting of a subset of nodes *V’* from *V* where *all* the edges linking the nodes of *V’* found in *V* are in the set *L’*.” We also added additional details on how graphlets can be used to compare networks in the Methods section, and S1 Appendix.

**[Reviewer #1]:** Why did you measure the dispersion of network topology by calculating the average pairwise distances between all networks of the same domain? Wouldn't be the variance a much more informative descriptor?

**[Response]:** This is an interesting point, which should now be better addressed by the new S1 Appendix Section S1.1. While it *would* be possible to evaluate a form of variance in DGCD between networks of the same domain (instead of the provided mean pairwise distance measure of dispersion) this would require projecting the DGCD first (to obtain a viable *mean* used in a variance calculation).

As depicted in Figure S4, the DGCD method provides pairwise DGCD between all networks (i.e., all networks supplied to the method). In other words, we only have available pairwise distance measures, and not explicit locations for where in space these networks occupy (in relation to all other networks). In order to evaluate the variance (instead of our mean pairwise distance dispersion metric), we would need to first determine the centroid of a group of points (i.e., the mean used in a variance calculation), which of course would require projecting these points in Euclidean space (or some other type of space). Although this is possible, it is difficult given that projecting *pairwise distances* is a heuristic process and dependent on the dimensionality of the projection. While we do ultimately project all pairwise distances between networks in 2-dimensional space (i.e., Figure 4), we do not rely on this projection for an estimate of dispersion. Since we calculate dispersion only on the average distance between all networks from a given domain or subgrouping (e.g., all networks that are Actor), we only rely on the pairwise DGCD values provided in the matrix rather than the distance that is projected in Euclidean space. Hence, we believe our dispersion metric is more accurate than a measure of variance.

In any case, we could also evaluate the variance in the pairwise DGCD between networks (where the mean used in the variance function would be the average DGCD distance). This would additionally provide a measure of dispersion around the mean DGCD for a specific network domain or subgrouping. We chose not to do this as it would simply be a measure of dispersion of dispersion and could inadvertently send the wrong message to readers. For example, using this metric we could end up with a very small value even when the average distance between networks is very large.

**[Reviewer #1]:** I do not understand how you go from the pairwise DGCD-13 between all networks and the multidimensional scaling represented in figure 4. I might miss that step, but you only provide a single sentence at the beginning of the results section: "The pairwise DGCD-13 between all networks was projected via multidimensional scaling" (line 154).

**[Response]:** Thanks for pointing this out as it is a problem if our steps are not clear. We have now explicitly covered this topic in Section S1.1 of the S1 Appendix. Generally, since multidimensional scaling (MDS) can project a dissimilarity matrix (where rows and columns represent specific networks and entries of the dissimilarity matrix represent pairwise dissimilarities between the corresponding networks), we simply took advantage of this property to visualize network dissimilarity. We have now noted and cited the function that was used to do this within the manuscript, on LN 207: “The pairwise DGCD-13 between all networks was projected via multidimensional scaling (MDS) (Borg and Groenen, 2005), also commonly known as principal coordinate analysis, using the MDS function in the Scikit-learn library (Pedregosa et al., 2011) of Python.” We have also shown how this was done within S1 Appendix Section S1.1.

**[Reviewer #1]:** Networks published in the same publication were built usually in different places on the same time scale using a standardized method to answer a specific question, and in most cases, for a single type of interaction. Therefore, we expect them to be more similar topologically than networks that were compiled from different authors with different motivations. For this reason results must be interpreted with caution: "networks from the same publication were more topologically similar, and thus publication had an even greater effect on the topology of networks than biology alone" (lines 182-183).

**[Response]:** You are indeed correct, and our mentioned statement wasn’t meant to reflect that publication trumped biology – but instead that networks from the same publication appear as less topologically heterogeneous as compared to any of the ecological subgroups (e.g., plant-pollinator networks), likely due to the fact that networks from the same publication are exposed to similar *biological and environmental drivers*, *sampling strategies*, and *network construction* *methods*. To fix this, we have removed this part of our results. Instead, we now say (starting on LN 245): “Nevertheless, although networks from the same publication were generally of the same ecological subgroup (i.e., most networks from a specific publication belonged to either ant-plant, host-parasite, plant-herbivore, plant-pollinator or seed-dispersal), networks from the same publication were more topologically similar than any single ecological subgroup.” We, however, feel that this thought could be better expanded upon in the discussion. Specifically, starting on LN 336, we have now written: “Consequently, it appears that publication has an even greater impact on the topology of species interaction networks than biological processes alone. This may occur since researchers of a given publication generally construct networks under parsimonious conditions (Brimacombe et al., 2022b), for example by observing and characterizing ecological communities across the same time duration [e.g., Trøjelsgaard et al. (2015)] or by classifying nodes across networks using the same protocol [e.g., Pereira Martins et al. (2020)], and thus inadvertently control for many sources of topological heterogeneity. Of course, biological effects are likely influencing the topology of all networks but they can be more easily obscured when analyzing networks across publications. It is then likely that controlling for the effect of publication can reduce unwanted topological heterogeneity between networks.”

**[Reviewer #1]:** Species interaction networks found in open databases are highly heterogeneous and we should pay more attention to the particularities of each network before performing any global analysis. I totally agree. Specifically, a careful consideration should be taken when comparing species interaction networks. But we could also see the glass as half-full and say that, even with the large heterogeneity in biotic and abiotic conditions, temporal and spatial scales, sampling effort and methodological approaches, there are some network properties that seem to pervade species interactions networks. DGCD is a global measure of the topological differences among networks, but I think that metric does not invalidate the existence of the same specific pattern that might be hidden under networks with different DGCD (e.g. nestedness). In fact, the suggestion you provide on controlling for the publication source of each network as a fruitful approach when reusing and analyzing species interaction networks is a powerful message (lines 261-263).

**[Response]:** This is an important point that we failed to mention. We have now added to our Discussion that there is documented evidence of some topological patterns in ecological networks which is not invalidated by our results. Specifically, on LN 387, we write: “Although we generally failed to find pervasive and coherent topology within species interaction networks created by different sets of researchers, we highlight that our results do not necessarily invalidate patterns others have found [e.g., high nestedness in plant-animal networks (Bascompte and Jordano, 2007)]. Instead, these patterns are perhaps true under strict conditions, such as controlling for the unwanted differences in topology between studies when reusing their networks.”

------------------------------------

**[Reviewer #2, Dr. Christian Mulder]:** Dear Authors,

My most sincere compliments for your outstanding revision and for the challenging topic chosen for your research. It is an extremely important issue in Food Web Ecology and I warmly welcome your work, very well done!

**[Response]:** We thank Dr. Christian Mulder for their enthusiastic appraisal of our manuscript!

------------------------------------
**[Reviewer #3, Dr. Adrian David González Chaves]:** I think is a relevant tropic and enjoy the fluidity while reading the work; the authors present an issue which is currently relevant and meant to be more so with time. Meta-analysis and synthesis analysis are become popular and needed and cautions about how to proceed with them are crucial (Mestre et al. 2022; Xing & Fayle 2021). Nonetheless, I feel that the main issue that the authors claim to tackle is not being fully address and could be explored with the data available, as they use a very extensive database. The following paragraphs are intended to rise the concern about how the authors explores the variability between networks and try to exemplify how could the fully answer the questions raised in the introduction.

**[Response]:** We thank Dr. Adrian David González Chaves for their thorough review. We also thank them for drawing our attention to Mestre et al. (2022) for which we meant to include as a reference but seemingly forgot to. Also, we have tracked all additional text in the manuscript by including them as orange. All unchanged text in the manuscript is written in black, and all of our responses (in this file) are written in blue.

We addressed each of their concerns in the following responses.

**[Reviewer #3]:** Authors main claim is that variability between network structure of ecological interaction is greater than other networks, e.g. social networks and microbial networks. Clearly watching from the supplementary material, the sizes of the ecological networks are inferior to any of the other networks considered (Table A1). Knowing that network size is fully influential on networks structure I wonder if this somehow accounted in the DGCD-13 metric considered.

**[Response]:** The size of networks (i.e., number of rows + number of columns of a network’s adjacency matrix or a network’s number of nodes) is not accounted for in the DGCD analyses. This is primarily because the original Authors of the DGCD method did not want to differentiate between networks based on the number of nodes since it would not be helpful to do so.

While we fully recognize that network size likely influences our results (just like it would any other type of network metric), we would like to point out that crime networks do have a similar size as species interaction networks (Table S1), but were significantly less topologically heterogeneous [e.g., 0.509 mean pairwise DGCD-13 for Minneapolis crime networks vs. 1.101 mean pairwise DGCD-13 for species interaction networks (Table 3)]. Moreover, we could distinguish between different types of crime networks (i.e., Chicago from Denver crime networks), and crime networks from other types of non-ecological interaction networks (Figure 4). Indeed, we specifically included crime networks for this reason, namely, to show that this method can still separate and identify small(er) networks. We have now added a paragraph in the Discussion (starting on LN 373) which covers this topic: “A limitation in our analyses was the use of small species interaction networks (e.g., <100 nodes). Since networks with a small number of nodes and edges are generally more difficult to classify than larger networks (Yaveroglu et al., 2014), we perhaps inadvertently increased the perceived topological heterogeneity of species interaction networks as compared to some of the non-ecological networks. Regardless, the crime networks we used were of similar size to species interaction networks (Table S1), but were less topologically heterogeneous (Table 3 and Figure 4). Clearly then, it was still possible to find topological consistency even in small networks, but less so when networks were both small and ecological. This suggests that the topological heterogeneity in species interaction networks created by different sets of researchers was due to more than just the difficulty of classifying small networks, but likely also from *biological and environmental drivers*, *sampling strategies*, and *network construction methods* classes of heterogeneity, which reused networks created by different sets of researchers are especially prone to. Importantly, this same problem of using small networks is also relevant when applying any other types of metrics to ecological networks, e.g., nestedness, modularity.”

As well, as we describe further in our responses, we did test if the size of species interaction networks contributed to differences in their mean pairwise DGCD distances/topological heterogeneity. Altogether, we find that regardless of size, species interaction networks generally have the same amount of topological heterogeneity.

**[Reviewer #3]:** Furthermore, the non-ecological networks seem to have a lot of standardization, less prone to the topological heterogeneity the authors expect to find in ecological networks.

**[Response]:** This is precisely the reason why we decided to include non-ecological networks in this manuscript: to show that other real-world networks tend to have significantly less topological heterogeneity than ecological ones. We believe this grounds the manuscript in reality since we can heuristically measure the amount of topological heterogeneity we would at least expect if ecological/species interaction networks were perhaps better standardized. To the best of our knowledge, this has not been done previously. We have now indicated this in our Introduction, starting on LN 73: “As most ecological networks do not have metadata regarding the conditions under which each network was created (i.e., their associated *biological and environmental drivers*, *sampling strategies*, or *network construction methods*) we could not partition topological heterogeneity across the heterogeneity classes. However, as we quantified the topological dispersion in non-ecological networks from different domains which are, to a large extent, not hampered by the three classes of heterogeneity, we could use these dispersion values to estimate the total amount of topological heterogeneity as a result of all three heterogeneity classes in species interaction networks.” We have also now stated why these non-ecological networks would have more standardizations (starting on LN 105): “We included non-ecological systems for comparison in our study given the strict definitions used to define their systems, thereby eliminating much of the *biological and environmental drivers*, *sampling strategies*, and *network construction methods* classes of heterogeneity that can strongly influence the topology of species interaction networks created by different sets of researchers. Here, “strict” refers to the high likelihood that the data for these non-ecological systems were recorded consistently using such definitions that their respective nodes/edges would more accurately and precisely reflect their intended purpose when implemented as a network, as compared to species interaction networks. Furthermore, we either built each non-ecological network ourselves (i.e., sports networks), or used those previously built by us [i.e., all non-ecological networks other than sports were obtained from Michalska-Smith and Allesina (2019)], thus ensuring appropriate data were used to build each non-ecological network. Indeed, the data used to build these networks came from specific databases for each domain (or subgroup within each domain, where subgroup refers to the different categories of a domain that networks represented, see Table 2). Moreover, as the data for each domain/subgroup of non-ecological networks came from the same database, if any class of heterogeneity were to influence their topology, the resulting heterogeneity would at least be consistent, and thereby reduce potential dispersion in measured topological heterogeneity. While undoubtedly the three classes of heterogeneity still influence non-ecological networks, for instance, due to the misidentification of nodes, we expected that these classes would be significantly less influential than those within species interaction networks. In particular, we expected large amounts of topological heterogeneity in available species interaction networks created by different sets of researchers resulted from the inconsistent ways ecological communities were translated into networks by the different sets of researchers. We expected this would have introduced inconsistent topology across species interaction networks thereby increasing the dispersion in measured topological heterogeneity.”

**[Reviewer #3]:** While only for Crime and Sport networks there are subgroupings (states and sport discipline) for the rest of the networks there are not categories, while for ecological networks there are either seasonal (already explored by the first author in another work), latitudinal mentioned in the literature cited, networks might have been gathered through different sampling method. Thus, it is expected that such variability will be found. The novelty would be whether the DGCD-13 methodology is good for assessing how much of the variability can be attributed to each topological heterogeneity class and not as a response of unwanted topological distances.

**[Response]:** Regarding the novelty aspect: while we recognize that others have pointed out the existence of different heterogeneities in ecological networks (some of the sources we have now added in response to Reviewer 1), since many ecological studies still do not attempt to control for such sources of variation and even recently continue to be published in high impact journals, we believe this is an important narrative that needs to be continued. We have highlighted this importance in our Discussion, starting on LN 300: “Importantly, even though *biological and environmental drivers*, *sampling strategies*, and *network construction methods* classes of heterogeneity are known to influence the topology of species interaction networks, they are nevertheless rarely acknowledged or appropriately controlled in ecological studies. This is especially problematic when reusing networks created by different sets of researchers since the influence of these classes are likely to vary considerably depending on the methods and approaches that different researchers use to create each network.” We also believe our novelty is in the framework we have designed and the methods we have used to quantify this heterogeneity.

Regarding subgroups in non-ecological networks: it is true that of the non-ecological networks, only crime (i.e., Chicago, Denver, Minneapolis, San Francisco, Washington), and sports (i.e., baseball, basketball, hockey) networks had unique subgroupings within their domain, as illustrated in Figure 4. However, there are *theoretical subgroupings* (i.e., subgroups that did not form subgroupings in Figure 4) within all other non-ecological networks. For instance, and as described in Table 2, “Actor networks” were composed of 20 different movie genres/subgroups (e.g., action, adventure, animation, etc.), and “Legislature networks” were composed of 4 different legislative bodies/subgroups (i.e., U.S. House, U.S. Senate, the U.N. General Assembly, European Parliament). Hence, it was possible for each of these subgroups to form its own subgroupings within Figure 4. But evidently, they did not have a distinct enough structure to do so, at least in the DGCD metric we used. We believe this is an important point that we failed to make, so we have added this in our Results, starting on LN 221: “Since every network’s domain was composed of multiple different subgroups (e.g., actor networks were made from action, adventure, . . . , western movie genres/subgroups, Table 2) each domain could have potentially formed their own distinct subgroupings within Figure 4 if they exhibited unique substructure like crime and sports networks.”

We contend, however, that non-ecological networks are also exposed to changes in their topologies across time/space (and of course other factors) rather than just species interaction networks. For instance, changes to voter ideologies in the legislative networks across space (e.g., between the U.S. House and the U.N. General assembly) likely contributed some topological heterogeneity. We thought this also important to include, so we have now written in our Discussion, starting on LN 285: “Indeed, the large difference in the amount of topological heterogeneity between species interaction networks and non-ecological networks may be attributed to these three classes of topological heterogeneity given that the non-ecological networks were created in a consistent way to try to eliminate much of their influence. For example, we built non-ecological networks using data attained from consistent *sampling strategies* (e.g., each sampled crime network represented a specific city and day of the year in 2016) and we used consistent *network construction* definitions when building the networks from the data (e.g., all interactions in crime networks always represented a type of crime occurring in a city’s specific neighborhood). This is not to say that non-ecological networks are devoid of their own sources of topological heterogeneity. For instance, differences in both voter sentiment across time and differences in the political landscape across space within the legislative networks (e.g., between U.S. House and U.N. General Assembly networks) likely contribute some topological heterogeneity. However, the *biological and environmental drivers*, *sampling strategies*, and *network construction methods* classes of heterogeneity seem to be accentuated in species interaction networks created by different sets of researchers as compared to the tested non-ecological networks.”

With regards to the comment on the ability of the DGCD method to determine the amount of topological heterogeneity due to each class of heterogeneity (i.e., *biological and environmental drivers*, *sampling strategies*, and *network construction methods*): we completely agree that this would be a very good use of resources. However, we have currently no way to do so (regardless of the methods we apply), since foremost the data required to do so are nonexistent. For example, while recent studies are now attempting to investigate how network topology is influenced by sampling time and sampling area (e.g., CaraDonna et al., 2021, Galiana et al., 2022), these studies still rely on reusing networks created by different sets of researchers and thus are hindered by the three classes of topological heterogeneity. We have now mentioned that we are unable to evaluate the amount of topological heterogeneity each class has on the topological dispersion of species interaction networks in our Introduction (starting on LN 72): “To measure topological heterogeneity, we evaluated the total dispersion in directed graphlet correlation distances between networks of the same domain (defined below). As most ecological networks do not have metadata regarding the conditions under which each network was created (i.e., their associated *biological and environmental drivers*, *sampling strategies*, or *network construction methods*) we could not partition topological heterogeneity across the heterogeneity classes. However, as we quantified the topological dispersion in non-ecological networks from different domains which are, to a large extent, not hampered by the three classes of heterogeneity, we could use these dispersion values to estimate the total amount of topological heterogeneity as a result of all three heterogeneity classes in species interaction networks.”

**[Reviewer #3]:** I don't about this metric, but I have some basic questions relating this metric. Is it possible to build a probability test over the values it generates? How do you known that the 76% of difference in variation reported doesn't occur by chance or if it is influenced for some extreme values within the 723 networks considered. For instance, and regarding the former comment, the size variability of the ecological networks considered are higher when compared to the other networks, so I strongly suggest incorporating a test that allow to consider such factor of variability, given this is present both for ecological and non-ecological networks. You consider the mean values to compare, but how much of the mean variability values are a result of the variance in network size. From what I understood the DGCD-13 analysis considers graphlets (or motifs), which are known to be influenced by the networks size, as it occurs with the networks structure (Milo et al. 2002 - Science: DOI: 10.1126/science.298.5594.824).

**[Response]:** To help reduce the possibility of the mean DGCD-13 being influenced by outliers/extreme values, we have now also performed the analysis using median values – and find very similar results. We now include this in our results section, starting on LN 226: “Of networks from the same domain or networks that had their own subgroupings within the MDS plot (Figure 4), species interaction networks had the largest mean pairwise DGCD-13 of 1.101—about twice as much as the set of legislation networks which was the next domain or subgrouping with the most topological dispersion (Table 3). This pattern also held when using median pairwise DGCD-13 (Table S2) and so mean DGCD-13 was not significantly influenced by outliers.” We recognize that this doesn’t fully solve the issue as outliers can still influence the median, but at least it is less influenced by outliers.

We also tested whether reducing network size variability reduced the DGCD values within species interaction networks. To do this, we ordered species interaction networks by size and grouped them into quartiles (based on size). We then used the first (smallest networks) and last (largest networks) quartiles which had drastically different network size variabilities (i.e., standard deviation of network size for first quartile=4.259, standard deviation of network size for last quartile= 217.844, respectively), and found that their mean DGCD-13 did not drastically differ (i.e., 1.0888 and 0.982, respectively) (Table S4). Hence, it does not appear that the variability in network size contributed significantly to DGCD-13 in species interaction networks. We have now added this in a new subsection to our S1 Appendix (i.e., S1.3.2): “It could have been the case that the larger variability in species interaction network size (i.e., number of nodes) caused its larger mean pairwise DGCD-13, as compared to other non-ecological networks which generally had less variability in network size. To test whether this was true, we divided species interaction networks into quartile groups (by network size) to reduce variability in size across a group. We then evaluated the mean pairwise DGCD-13 between the smallest networks (i.e., those within the first quartile) and the largest networks (i.e., those within the fourth quartile) (Table S4). We removed any networks sourced from the same publication, to eliminate the effect of publication in this analysis. If multiple networks were sourced from the same publication within a quartile, we only kept a single network from that publication. A single network was chosen using a simple criterion: first, we kept the network with the largest size. If more than one network from the same publication had the same size, we then chose the network with the highest connectance value. If again, more than one network had the same connectance, we chose the network with the greatest number of rows in its adjacency matrix.

Altogether, we found that the standard deviation in network size for the first quartile (representing the smallest networks in our analyses) was considerably smaller in relation to its mean network size as compared to the fourth quartile (representing the largest networks in our analyses). Specifically, the first quartile had a standard deviation in network size of 4.259 and mean network size of 18.544 while the fourth quartile had a standard deviation in network size of 217.844 and mean network size of 170.652 (Table S4). However, the first quartile had a larger mean pairwise DGCD-13 than the fourth quartile (1.088 vs. 0.982, respectively) which would not have been expected if the variability in network size influenced mean pairwise DGCD-13. Hence, it does not appear that the variability of network size strongly affected the mean pairwise DGCD-13 in species interaction networks.” However, we would also like to remark that species interaction networks are not the only networks with significant standard deviation in their network size. For instance, Actor networks also had a large variation in their number of rows (standard deviation=8371.04) and columns (standard deviation=1391.519) (Table S1), but had significantly less variability in their structure and formed a coherent group in the MDS plot (Figure 4). Hence, variation in network size is not solely a species interaction network feature.

With regards to the pairwise distances being influenced by chance: we cannot say for sure the degree to which network dissimilarity is due to chance. It is entirely possible that much of the network structure is due to someone observing a chance interaction and recording it as an interaction in the network. In fact, we agree that this likely does contribute to large differences between network topologies that we observe. And for this very reason, we used real non-ecological networks to compare to (rather than relying on significance tests) to see what sort of real topological heterogeneity would be expected if species interaction networks created by different sets of researchers were perhaps defined in a more consistent manner. Nevertheless, while it is common to compare empirical networks to null model networks, like motif analyses, to get an estimate of chance, this is actually, as we argue, a limitation of motifs since it relies on the assumption that a null model is well-posed. Depending on which null-model is selected, networks can either appear random or be found to have different degrees of non-random structure. We have now added this to our Methods, starting on LN 135: “In ecology, the most adopted subgraph technique is based on motifs. Generally, for a graph *G* composed of a set of nodes *V* and a set of links *L*, denoted as *G*(*V*, *L*); a motif of *G* is a subgraph *G’*(*V’*, *L’*) with a subset of nodes *V’* from *V* where any edges linking the nodes of *V’* found in *V* are contained in *L’*. Motif interpretation is highly dependent on the choice of a network null model since motifs are defined as small partial subgraphs that are over-represented in the real network compared to a chosen network null model (Pržulj et al., 2010; Yaveroglu et al., 2014). As a consequence, motifs have been criticized for possibly relying on ill-posed null models as a basis for significance testing (Artzy-Randrup et al., 2004).

To overcome the null model limitation of motifs, we instead adopted the subgraph technique of directed graphlet correlation distance (DGCD) (Sarajlić et al., 2016) to characterize the topological differences between networks. Generally, DGCD evaluates network pairwise dissimilarity without relying on a network null model, and does so by quantifying differences in the associations between the appearance of directed graphlets (Figure 3) within a given network to those of another network.” Thus, while it doesn’t give us a measure of chance, it does provide us with a more accurate measure of network similarity and dissimilarity.

**[Reviewer #3]:** In the abstract you mentioned: "to evaluate the degree to which this topological heterogeneity is present in available ecological networks, …". I feel this is not fully explored from your data and could be explored with a statistical test. I wonder whether you could use the mean DGCD-13 value as a starting comparison point, to compare how much each network deviates from it, and then test how much of each heterogeneity class could explain the variability found. Then you would have an attributable value to how much of the biological, sampling or network construction is predicting the network structure variability. The work you refer to, where you used the same data, does this (Brimacombe et al 2022) using seasons, but in that case, you are not looking to network structure variability as you calculated here. Meaning you have information on network size and temporal elements of the studies to assess how much of the higher variability found can be attributed to each topological feature and address what is mentioned in rows 39 to 41. Because from what I understood, the quantification that you say is going to be address in rows 67 to 70, is not really undertaken. You seem to infer the role of the topological heterogeneity from comparing variability among researchers and between ecological networks and other networks, and not through an integrated analytical approach.

**[Response]:** As per our comment about disentangling the amount of topological heterogeneity due to each of the three classes of topological heterogeneity (i.e., *sampling strategies*, *biological and environmental drivers*, and *network construction methods*): we do believe that this is a necessary step in understanding how to properly reuse networks created by different sets of researchers. However, to attempt to resolve this issue would require a lot of new data from the creator of each network since most networks in these databases do not have the information/metadata on how each network was created. For example, we don’t have information regarding the duration and frequency of observation used to characterize each community as a network, so we can’t test such a relationship as temporal elements on network topology. To better emphasize that we are unable to do this *at the outset*, we have now added a sentence to the Introduction that detailed what we had done in our manuscript. Specifically, starting on LN 72: “To measure topological heterogeneity, we evaluated the total dispersion in directed graphlet correlation distances between networks of the same domain (defined below). As most ecological networks do not have metadata regarding the conditions under which each network was created (i.e., their associated *biological and environmental drivers*, *sampling strategies*, or *network construction methods*) we could not partition topological heterogeneity across the heterogeneity classes. However, as we quantified the topological dispersion in non-ecological networks from different domains which are, to a large extent, not hampered by the three classes of heterogeneity, we could use these dispersion values to estimate the total amount of topological heterogeneity as a result of all three heterogeneity classes in species interaction networks.” Nevertheless, we do think it is important to discuss future opportunities to investigate how each class of heterogeneity could influence topology. We have now added recommendations for authors of networks to include such metadata information in order to perform these investigations (see comment below for a more detailed response).

We could, of course, attempt to try and find such relationships between the amount of heterogeneity in network topology in some instances (as you indicate). However, often times there is no statistically significant relationship found between network topology and sources of topological heterogeneity, such as seasonality (which was found in our referenced paper). Hence, we wouldn’t always be able to reliably identify which topological heterogeneity was due to which class/source of heterogeneity.

**[Reviewer #3]:** Probably why your discussing and conclusion are seems to be calling attention to considering this higher variability when implementing meta-analysis, but for me it suggests that give the existence of the high variability there is space for testing how much other factors (or grouping) explain the variability found (as you seem to have done for the other referenced research). Again, could you not create a model to estimate how much of the variation found in the Ecological networks can be attributed to each of the topological categories mentioned? How is that you can disentangled between each category? To illustrate the need to take this into account, and how to do so.

**[Response]:** This is an interesting way to flip it: instead of trying to control for the classes of heterogeneity (given the appropriate metadata to do so), one would also be able to study how much each class contributes to topological heterogeneity. We have now added this in our Discussion, starting on LN 354: “In particular, by providing detailed network metadata, including information on relevant *biological and environmental drivers*, *sampling strategies*, and *network construction methods*, authors of the networks can help others understand the specific conditions under which each network was created. Additionally, given the recent developments of composite methods designed to estimate sampling sufficiency for ecological networks [e.g., Casas et al. (2018)], authors of species interaction networks could also calculate this metric or provide the information to do so to check if communities have been sufficiently sampled. Then, beyond controlling for sources of topological heterogeneity (e.g., node taxonomy), researchers reusing these networks could also control for sampling sufficiency which is another means to improve network commensurability. Given appropriate metadata, researchers could also study how each class of heterogeneity influences the topology of species interaction networks, rather than the totality of topological heterogeneity as we have done here.”

Regarding the modeling comment: since we are unable to determine the amount of topological heterogeneity that each class contributes to network structure, we are unable to model it. We believe the only way to effectively disentangle this problem, would be to create networks from scratch (via in situ observation) with the primary goal to try and decipher how each class of heterogeneity influences network topology (e.g., by holding all but one classes/sources of heterogeneity constant, and letting one class/source vary). This, of course, has rarely (if at all) been done. While we acknowledge that similar tests have been attempted, e.g., recent studies are now attempting to investigate how network structure is influenced by sampling time and sampling area (e.g., CaraDonna et al., 2021, Galiana et al., 2022), these studies still rely on reusing networks created by different sets of researchers and thus are hindered by the three classes of topological heterogeneity. Hence, studying how network topology is influenced by each class of topological heterogeneity would likely consume multiple papers (such as the described one of CaraDonna et al. (2021) for just time duration) and require new data to do so.

------------------------------------
**[Reviewer #4]:** I have read the manuscript PBIOLOGY-D-22-01700R1 by Brimacombe et al. with much interest. The authors evaluate the topological heterogeneity of ecological networks, comparing ecological networks to "non ecological" networks, and by assessing the heterogeneity of networks from ecological studies that have one network vs studies that have multiple networks. The authors found that the topology of ecological networks are much more heterogenous than "non-ecological "networks, and that interaction networks from the same publication are much more topologically similar to each other than interaction networks that come from the same study. They suggest that three different sources are likely to be the main causes of the heterogeneity they found in ecological networks, namely: sample strategy, bio-ecological drivers, and how networks are constructed. The question tackled by the authors is very interesting and important, given that in the past years, ecologists have tried to produce syntheses and tried to understand biogeographical patterns in species interaction networks amassing data from distinct publications, ignoring possible flaws with the available data. The authors also suggest some possibilities to deal with this problem. The results are interesting, and I believe the manuscript is an important contribution to the field of ecological networks. Nonetheless, I do have some concerns, that I think it should be addressed and some minor requests for change and/or suggestions, that hopefully should help to improve the manuscript and not take much time.

**[Response]:** We thank the reviewer for taking the time to review our manuscript, and we are especially glad to hear that our manuscript is of interest. We also appreciate the reviewer pointing out missing metrics and sources of heterogeneity not previously included in our framework (e.g., sampling sufficiency, interaction frequency, respectively) for which we have now included. We have tracked all additional text in the manuscript by including them as orange. All unchanged text in the manuscript is written in black, and all of our responses (in this file) are written in blue.

We have responded to each of their concerns in the following paragraphs.

**[Reviewer #4]:** Although I appreciate the efforts of the authors to compile and amass this amount of data, I was not convinced of some of the assumptions regarding the "non ecological networks", that make them a significant baseline to estimate the topological heterogeneity of ecological networks. The authors claim that ecological networks are hampered by biological, environmental drivers, sampling strategy, and/or network construction, and that the "non ecological" networks are not hampered by these sources of heterogeneity. However, the authors do not provide any evidence of references that can support this fundamental claim of the manuscript. Although, I do not have much familiarity with these kind of data, I found it hard to believe that these data sets don't have sources of biases or are not hampered by problems related to sampling sufficiency. For instance, crime reports are known to be biased, and I guess this could have an impact, I imagine that both author and actor networks will depend on the database being used, plus, they might share the same problem with ecological networks in regard to the misidentification of nodes, due to homonymous authors/actors, etc. I do not understand how microbiome networks were considered as "non"- ecological networks. Why are they not hampered by biological or environmental drivers, and how they can serve as the basis for you comparison here? Most importantly, microbiome sampling strategy also varies a lot as "ecological" networks do; e.g., gut microbiome networks can be constructed based on samples collected from feces, mucosal biopsy, intestinal fluid, etc. or come from different parts of the gastrointestinal tract.

**[Response]:** This is an error on our part since we failed to highlight why non-ecological networks would have more consistency in their topology (compared to ecological/species interaction networks). Overall, we do not mean to say that non-ecological networks are exempted from the listed classes of heterogeneity (i.e., *biological and environmental drivers*, *sampling strategies*, and *construction methods*). As you rightfully point out, non-ecological networks do have instances of missing nodes/edges just like species interaction networks (e.g., crime networks likely have missing reported crimes) but yet we still find that species interaction networks appear to have a lot more heterogeneity. Because non-ecological networks do have more consistencies but are still subject to some heterogeneity is exactly why we decided to include real networks: to show how networks which do have some problems could look like in terms of their topological heterogeneity. We have now explicitly written that non-ecological networks have their own problems in the Discussion of our manuscript (starting on LN 285): “Indeed, the large difference in the amount of topological heterogeneity between species interaction networks and non-ecological networks may be attributed to these three classes of topological heterogeneity given that the non-ecological networks were created in a consistent way to try to eliminate much of their influence. For example, we built non-ecological networks using data attained from consistent *sampling strategies* (e.g., each sampled crime network represented a specific city and day of the year in 2016) and we used consistent *network construction* definitions when building the networks from the data (e.g., all interactions in crime networks always represented a type of crime occurring in a city’s specific neighborhood). This is not to say that non-ecological networks are devoid of their own sources of topological heterogeneity. For instance, differences in both voter sentiment across time and differences in the political landscape across space within the legislative networks (e.g., between U.S. House and U.N. General Assembly networks) likely contribute some topological heterogeneity. However, the *biological and environmental drivers*, *sampling strategies*, and *network construction methods* classes of heterogeneity seem to be accentuated in species interaction networks created by different sets of researchers as compared to the tested non-ecological networks.” However, we do not have any evidence, other than the results we find, that the non-ecological networks presented here would definitely have less topological heterogeneity – since we constructed them ourselves [or have constructed them in previous publications, i.e., Michalska-Smith and Allesina (2019)].

Nevertheless, we highlight that the non-ecological networks used in our manuscript were constructed by us using only specific databases for specific domains/subgroupings (the exact sources of the datasets for which we used to construct the networks are now referenced in the metaData.csv file). Consequently, these networks were constructed with at least constant methods that can eliminate much of the heterogeneity you refer to. In other words, even if there were some misidentifications in nodes/edges, there would at least be continuity in these misidentifications across non-ecological networks of a given domain/subgrouping – which would *consistently* introduce a systematic source of topological heterogeneity. This compares very differently to ecological networks where there are no consensus’ on how nodes/edges are defined, leading to *inconsistent* structure and thus large amounts of topological heterogeneity. We have added this to the Methods section (starting on LN 105): “We included non-ecological systems for comparison in our study given the strict definitions used to define their systems, thereby eliminating much of the *biological and environmental drivers*, *sampling strategies*, and *network construction methods* classes of heterogeneity that can strongly influence the topology of species interaction networks created by different sets of researchers. Here, “strict” refers to the high likelihood that the data for these non-ecological systems were recorded consistently using such definitions that their respective nodes/edges would more accurately and precisely reflect their intended purpose when implemented as a network, as compared to species interaction networks. Furthermore, we either built each non-ecological network ourselves (i.e., sports networks), or used those previously built by us [i.e., all non-ecological networks other than sports were obtained from Michalska-Smith and Allesina (2019)], thus ensuring appropriate data were used to build each non-ecological network. Indeed, the data used to build these networks came from specific databases for each domain (or subgroup within each domain, where subgroup refers to the different categories of a domain that networks represented, see Table 2). Moreover, as the data for each domain/subgroup of non-ecological networks came from the same database, if any class of heterogeneity were to influence their topology, the resulting heterogeneity would at least be consistent, and thereby reduce potential dispersion in measured topological heterogeneity. While undoubtedly the three classes of heterogeneity still influence non-ecological networks, for instance, due to the misidentification of nodes, we expected that these classes would be significantly less influential than those within species interaction networks. In particular, we expected large amounts of topological heterogeneity in available species interaction networks created by different sets of researchers resulted from the inconsistent ways ecological communities were translated into networks by the different sets of researchers. We expected this would have introduced inconsistent topology across species interaction networks thereby increasing the dispersion in measured topological heterogeneity.”

With regards to inconsistencies within the topology of microbiome networks: the data used to construct microbiome networks were from a single and highly cited NIH Human Microbiome project with consistent sampling and sequencing techniques. Hence, there were no differences in how data were collected between microbiome networks. We have now mentioned this, starting on LN 89: “We classified microbiome networks as non-ecological since, among other properties, they were not built using observational data (instead, for example, by swab and subsequent RNA sequencing), and had concrete definitions for their edges/nodes (i.e., locations on the human body where a bacterial operational taxonomic unit was found)—two stark features that differ from species interaction networks (our ecological networks). See Aagaard et al. (2013) for a thorough description of how patients were selected, and operational taxonomic units were sampled, which were the data we used to build the microbiome networks. Although microbiome networks could be considered ecological, we believed that their topological heterogeneity would more resemble non-ecological networks and thus grouped them accordingly.” We think it is fair to say that RNA sequencing and a highly selective patient screening (as is required with a human-centric study) would be significantly less influenced by the three classes of heterogeneity as compared to the observational species interaction studies created by different sets of researchers which do not provide such comprehensive material for their sampling strategies. However, we also now highlight why we expect species interaction networks to be highly topologically heterogeneous in our Discussion as referenced in the first paragraph of this response.

**[Reviewer #4]:** I also wonder, even if those assumptions related to the "non-ecological" networks hold, if the variability found here could not be due to the fact that bipartite networks are more variable than unipartite networks, which form your entire reference system? Is there any evidence that can support that?

**[Response]:** The non-ecological networks used in our manuscript are in fact bipartite networks as indicated starting on LN 85: “A total of 3476 bipartite networks were used in this study (see Table 2 for a description of all networks and their domains). Of the non-ecological bipartite networks included in our analysis, 1830 were of the crime domain, 109 were of the journal domain, 245 were of the legislature domain, 172 were of the actor domain, 194 were of the sports domain, and 203 were of the microbiome domain.” We have now added “bipartite” to the second sentence to further emphasize this point. Moreover, we defined each domain’s two sets of nodes (i.e., the two partitions of nodes that make up the bipartite networks) in Table 2. Thus, there is no additional topological heterogeneity in species interaction networks as compared to non-ecological networks due to non-ecological networks being unipartite.

**[Reviewer #4]:** Most importantly, I think one of key aspect missing here is the problem related to sampling sufficiency/completeness, which is a fundamental aspect of sampling theory that is largely overlooked by network ecologists. There is no way to move forward, unless network ecologists start to pay attention and report that. Although this has been a pivotal problem in both population and community ecology, network ecologists seem to get a free pass, not accounting for it or using inappropriate methods to estimate sampling sufficiency. However, most networks published to date are likely to be under-sampled, and to make things more complicated, evidence suggest that sampling sufficiency is metric specific, even for the same dataset (Casas et al. 2018. Assessing sampling sufficiency of network metrics using bootstrap. Ecological Complexity 36 268-275), which means that, even if networks have been properly sampled in order to estimate a specific topological property, they may not be commensurable, if original studies were designed to estimate different topological properties.

**[Response]:** This is an important point that we had not appropriately addressed in our manuscript so thank you for bringing it up. We considered sampling sufficiency as related to “sampling strategy” covered by the temporal elements and spatial elements of study (from our classes of heterogeneity framework). Our thinking was that the more time/area sampled (duration, extent, resolution, or time-interval, as covered by the temporal and spatial elements of the framework) to capture a community as a network, the better it will be represented by a network (up until the point when the network is sufficiently sampled). In other words, we do not consider sampling sufficiency as a source of topological heterogeneity, as we define them in our manuscript, but we now consider sampling sufficiency as another means to measure the quality of one’s network data. We have now indicated this sentiment starting on LN 310 of the Discussion: “Furthermore, related to variations in *sampling strategies*, networks may also vary in their sampling sufficiency (Casas et al., 2018). Insufficiency can occur when the sampling design does not match the biology of the community and can make networks incommensurable even when networks are built using the same sampling strategies.” We have also added to our Discussion that authors of networks could provide sampling sufficiency metrics for their networks to help with commensurability (starting on LN 357): “Additionally, given the recent developments of composite methods designed to estimate sampling sufficiency for ecological networks [e.g., Casas et al. (2018)], authors of species interaction networks could also calculate this metric or provide the information to do so to check if communities have been sufficiently sampled. Then, beyond controlling for sources of topological heterogeneity (e.g., node taxonomy), researchers reusing these networks could also control for sampling sufficiency which is another means to improve network commensurability.”

We like your point about how metrics are differently affected by sampling sufficiency, and so in our Discussion, we have written (starting on LN 345): “We do, however, strongly caution those that only attempt to account for the publication source of each reused network. Similar to how different network metrics are sensitive to different amounts of sampling sufficiency (Casas et al., 2018), the strong similarity between networks from the same publication may be more or less relevant when investigating network structure using other metrics.”

We do note however, that while sampling sufficiency could help identify and separate well-sampled networks from not well-sampled networks, as we advocate in the manuscript, there is still a lot that can be different between networks (e.g., node taxonomy, amount of area used to capture a community as a network, species of interest, type of ecological system) which could make them incommensurable.

**[Reviewer #4]:** There has been some great efforts in the past years in order to create appropriate methods to estimate sampling sufficiency for ecological networks, see for instance: Casas et al. (2018), Jordano (2016. Sampling networks of ecological interactions. Funct Ecol, 30: 1883-1893) and references therein.

**[Response]:** We had referenced Jordano (2016) in the manuscript but had not come across Casas et al. (2018). We agree that it’s a good paper and have now included this citation when referencing difficulties of comparing networks. Moreover, we like this idea that sampling sufficiency provides another means to evaluate network commensurability. We have added this sentiment to our Discussion (starting on LN 357): “Additionally, given the recent developments of composite methods designed to estimate sampling sufficiency for ecological networks [e.g., Casas et al. (2018)], authors of species interaction networks could also calculate this metric or provide the information to do so to check if communities have been sufficiently sampled. Then, beyond controlling for sources of topological heterogeneity (e.g., node taxonomy), researchers reusing these networks could also control for sampling sufficiency which is another means to improve network commensurability.”

**[Reviewer #4]:** At last, different from community ecology, recording pairwise species interaction demands that network ecologists account for species-specifics detection probability, and link(interaction)-specific detection probability. I wonder why the authors did not consider analytical solutions, such as hierarchical modeling as a possible solution form this problem, since it can account for detection probabilities, for nodes and link and account species misidentification. I like the direction given by the authors in terms of using studies as some sort of random effect, however the solution presented here will not suffice for the problems exposed in the manuscript, if authors of original studies do not account for sampling sufficiency and problems relate to node/link detection rates.

**[Response]:** We had not thought of doing analytical solutions to understand how each class of heterogeneity influences network topology (although the possibility for it could perhaps be many other papers), for two reasons. First, the networks available to us (i.e., those that are open networks) often have no metadata which would provide much of the necessary information needed to study such processes. Second, our target audience were those who are performing simple regression models using these available networks. So, we wanted to simply show how the large amounts of topological heterogeneity in species interaction networks can make finding patterns in species interaction networks somewhat of a “finding a constellation amongst the stars”. Hence, our first step was to recognize the issue of reusing networks created by different sets of researchers without consideration of the three listed classes of topological heterogeneity, rather than to provide a solution. We agree though, that this next step is an essential one to take, namely, to find effective ways to reuse network data.

As our target audience were those performing network analyses by reusing networks created by different sets of researchers (rather than those constructing the networks), it would be very difficult (if not impossible) for them to determine detection probabilities for each species/link/etc. for each network. As we have mentioned, metadata regarding, for example, the amount of time and area used to construct each network, is missing for almost all networks which is often used to estimate such measures of misidentification/other problems with observational data (as is required in say <https://www.nature.com/articles/s41467-021-24149-x> for using Bayesian statistics to estimate network structure from noisy observational studies). We have now mentioned why we were unable to evaluate the amount of topological heterogeneity as a result of each class of heterogeneity (starting on LN 72): “To measure topological heterogeneity, we evaluated the total dispersion in directed graphlet correlation distances between networks of the same domain (defined below). As most ecological networks do not have metadata regarding the conditions under which each network was created (i.e., their associated *biological and environmental drivers*, *sampling strategies*, or *network construction methods*) we could not partition topological heterogeneity across the heterogeneity classes. However, as we quantified the topological dispersion in non-ecological networks from different domains which are, to a large extent, not hampered by the three classes of heterogeneity, we could use these dispersion values to estimate the total amount of topological heterogeneity as a result of all three heterogeneity classes in species interaction networks.”

We agree sampling sufficiency is important topic, but for which we did not talk about in our previous manuscript, and so we have now included it as a Discussion point (see above responses).

**[Reviewer #4]:** L71-72. I would suggest rephrasing this sentence; as it gave me the impression that the authors were going to partition the heterogeneity among these three sources, which is not the case here. I think other readers would build the same expectation.

**[Response]:** This was a problem that we had thought of before, but we were hoping that we resolved it. Clearly not! We have now explicitly stated that we did not identify the amount of topological heterogeneity as a consequence of each class in the reference paragraph (now LN 72): “To measure topological heterogeneity, we evaluated the total dispersion in directed graphlet correlation distances between networks of the same domain (defined below). As most ecological networks do not have metadata regarding the conditions under which each network was created (i.e., their associated *biological and environmental drivers*, *sampling strategies*, or *network construction methods*) we could not partition topological heterogeneity across the heterogeneity classes. However, as we quantified the topological dispersion in non-ecological networks from different domains which are, to a large extent, not hampered by the three classes of heterogeneity, we could use these dispersion values to estimate the total amount of topological heterogeneity as a result of all three heterogeneity classes in species interaction networks.”

**[Reviewer #4]:** L121. The word "best" is duplicated in this sentence.

**[Response]:** Woops. We have now removed a single “best”, so the sentence now reads: “We used DGCD in our study since recently Tantardini et al. (2019) found that this method performs best at characterizing and distinguishing between networks of different domains.”

**[Reviewer #4]:** L199-202. That is assuming that networks published in each study has been properly samples and that topological estimators have reached some asymptotic property, which is unlikely. The problem here is that poorly, under- sampled networks are published, and then compiled together in syntheses/biogeography studies.

**[Response]:** Yes! And we would even go further to suggest that we need to be mindful of the amount of area, target species, target edges, …. which should also be considered when comparing networks created by different sets of researchers (as listed in our three classes of heterogeneity from our framework). However, these classes/sources of heterogeneity are often ignored when performing meta-analysis type studies with these networks. We have referenced the necessity to have quality data when collecting large amounts of data in the same paragraph that you reference: “However, using the largest set of bipartite species interaction networks available (*n* = 723), we illustrate how large amounts of topological heterogeneity (via the mean pairwise DGCD-13, Table 3) and consequently uncertainty exists in the topology of species interaction networks created by different sets of researchers, confirming that more data is not always better when biases are present (Dietze, 2017).”

Nevertheless, even with equal amounts of time/area/node resolution/etc., networks may not accurately represent their community since they have not been sufficiently sampled (as you indicate). As such, and in response to those comments above, we have also recommended the consideration of sampling sufficiency as an additional means to consider only those networks that best represent their community (i.e., networks with adequate sampling sufficiency).

**[Reviewer #4]:** L154: What is the exact method used for the MDS? Principal Coordinates?

**[Response]:** We should have been more explicit with this. Yes, the form of MDS we used was principal coordinates analysis. We have now mentioned this in the manuscript (starting on LN 207): “The pairwise DGCD-13 between all networks was projected via multidimensional scaling (MDS) (Borg and Groenen, 2005), also commonly known as principal coordinate analysis, using the MDS function in the Scikit-learn library (Pedregosa et al., 2011) of Python.”

**[Reviewer #4]:** L174. What is the average and/or range of the number of networks in publications with multiple networks? Could be that the small number of networks within a publication cause this small variability for the "multiple networks per publication grouping? Once again poor sampling sufficiency for this multiple networks could also be the factor influencing the results here. Is there any evidence that sampling sufficiency change between these two groupings for a specific metric?

**[Response]:** In S1 Appendix, we list the number of networks that each publication provides and the corresponding DGCD between networks of those publications (Table S3 for DGCD-13, and Table S8 for DGCD-6). This table has also been referenced in the manuscript when talking about publications that provide more than a single network (starting on LN 196): “Additionally, we tested whether species interaction networks created by the same set of researchers (i.e., networks sourced from the same publication) were more topologically similar than networks not sourced from the same publication, see Table A3 for a list of publications that provided more than a single network.” Using this table, we find that the average number of networks a publication with multiple networks provides is about 8.40.

The way we calculated the mean DGCD values (for networks that provide more than a single network) is somewhat independent of the number of networks a publication provides. It is simply the mean pairwise DGCD values between networks from the same publication, averaged (weighted by the number of networks per publication) across publications. Nevertheless, we have now additionally evaluated whether publications that provide fewer networks (but more than one) had lower mean DGCD values than publications that each provided a greater number of networks (Figure S6 and Section S1.3.1 in S1 Appendix). We observe that there isn’t much of a difference between the number of networks a publication provides and its mean DGCD value.

Certainly, sampling sufficiency is likely influencing our results. Moreover, the networks we use are binary (not weighted) and so the sufficiency metric proposed is not necessarily appropriate. Of course, we could attempt to control for sampling sufficiency (for the different metrics of sufficiency), but our goal for this manuscript is to simply show the large amounts of topological heterogeneity in species interaction networks created by different sets of researchers, and how the many sources of topological heterogeneity make comparing networks a difficult task. Although, given the many metrics for sufficiency, it may be fruitful to investigate this further and to determine which metrics are useful.

**[Reviewer #4]:** L199-210. Once again, assuming that each and every network has reached sampling sufficiency, for the problem at hand. See comments above on metric specific sampling sufficiency.

**[Response]:** While we agree with your sentiment, it is not the only thing affecting networks – even if networks had effective sampling sufficiency – there could be other issues, including lack of commensurability in the taxonomy of nodes (between or within networks), the different amounts of the area used to characterize communities as networks, the different types of interactions that edges represent, … that are not effectively captured by sampling sufficiency type metrics. In other words, sampling sufficiency may say a set of networks are comparable even though these listed problems still exist. We have now referenced that these metrics do not necessarily control for the different sources of topological heterogeneity (in the same paragraph): “While some metrics, including sampling intensity and effort, have previously been used to control for biases and sources of topological heterogeneity in species interaction networks (Brimacombe et al., 2022a), these controls do not effectively account for all sources of heterogeneity (e.g., differences in node taxonomy across networks) or when using different topological metrics (e.g., modularity, nestedness). Hence, careful consideration, beyond a single metric of control, is required when deciding which networks to include in one’s analyses, so that the majority of topological differences measured between species interaction networks are a result of the ecological process-of-interest and not from confounding factors.” However, as we described in some of our other responses (and addressed in the manuscript), sampling sufficiency is another metric that can be used to assess the quality of one’s network data.

**[Reviewer #4]:** L 259. Standardizing sampling time, does not guarantee that sampling sufficiency is reached given that detection rates are note homogeneously distribute across space… Species specific detection rates and iteration specific detection rates can between habitats.

**[Response]:** You are correct: there are likely a very large number of sources of topological heterogeneity that need to be considered for networks to truly be commensurable. But it certainly is one source of heterogeneity that is important to consider (i.e., are networks built over a time scale of months or a time scale of years?). There is some evidence to suggest patterns of network topology vary consistently with duration (CaraDonna et al. 2021: Seeing through the static: the temporal dimension of plant–animal mutualistic interactions), although these analyses do depend on reusing networks created by different sets of researchers, so other sources of heterogeneity could also be influencing their results. Nevertheless, as per your comment, we have now included mention of specific detection rates in our framework (both in Figure 1 and Table 1).

**[Reviewer #4]:** L267: Maybe most importantly here, is providing sampling sufficiency for the parameters that were investigated.

**[Response]:** It is true that sampling sufficiency could help assess commensurability between reused networks created by different sets of researchers (along with the other listed sources/classes of heterogeneity from our framework). We have now referenced sampling sufficiency as a tool that authors of networks could include in their metadata, as a means for others to measure commensurability of reused networks in their analyses (starting on LN 357): “Additionally, given the recent developments of composite methods designed to estimate sampling sufficiency for ecological networks [e.g., Casas et al. (2018)], authors of species interaction networks could also calculate this metric or provide the information to do so to check if communities have been sufficiently sampled. Then, beyond controlling for sources of topological heterogeneity (e.g., node taxonomy), researchers reusing these networks could also control for sampling sufficiency which is another means to improve network commensurability.”

**[Reviewer #4]:** Fig 1 and table 1 should or could also depict the fact that species specific detection probabilities, and interaction detection probabilities vary.. so even if a researcher have the same sampling strategy across space and time, may not be a sufficient strategy to deal with the problem.

**[Response]:** We have now included “interaction frequency” as a source of topological heterogeneity within both Figure 1 and Table 1, to emphasize your point. Specifically, in Table 1, we describe this as: “Number of interaction events an organism has influences the probability interactions are recorded in a network. (E.g., topological differences in networks when cryptic interactions are included.)”
