# Supplementary material for: Shortcomings of reusing species interaction networks created by different sets of researchers
Source: PLoS Biol. 2023 Apr 3;21(4):e3002068. doi: 10.1371/journal.pbio.3002068 (PMC10101633; doi:10.1371/journal.pbio.3002068)
Supplement: Attachment — Submitted filename: Response_to_reviewers_March_2.docx [file pbio.3002068.s004.docx]

**Reviewer #1:**

The main points raised in my previous review have been satisfactorily addressed. I just have a couple of very minor comments:

We thank Dr. Miguel A. Fortuna for their comments. In response, we have made additional changes to the manuscript and appendix, which we coloured as magenta. We note that the previous revisions in the manuscript remain coloured as orange.

1) I am wondering why you computed DGCD-13 instead of DGCD-6 (I am aware you reported DGCD-6 as well in the Appendix). You mention in Fig.3  that "Note that for the directed bipartite networks used in this study, only graphlets G0, G2, and G3 appear". Then, a node can occupy only 6 orbital positions (0, 1, 5, 6, 7, and 8). Why did you use 13 orbits to compute DGCD? You seem to justify this decision by saying: "According to these direction definitions imposed on the networks, only graphlets G0, G2, and G3 could appear although all six graphlets and 13 orbits were used for better visualization" (lines 184-185). I would appreciate a much informative explanation.

We chose to present DGCD-13 in the main manuscript (as opposed to DGCD-6) because it produced a more resolved multidimensional scaling plot (Figure 4 vs. Figure S7) while still yielding similar results as DGCD-6. We recognize that this wasn’t explicitly stated in the manuscript, so starting on LN 184 we now write: “According to these direction definitions imposed on the networks, only graphlets *G_0_*, *G_2_*, and *G_3_* could appear although all six graphlets and 13 orbits were used for better visualization—specifically Figure 4—but see S1 Appendix Section S1.4 for subsequent analyses using only the six orbits from graphlets *G_0_*, *G_2_*, and *G_3_*, termed DGCD-6. Nevertheless, we note that the results presented in this article for DGCD-13 agree with those presented in the S1 Appendix using DGCD-6.”

We have also now stated more precisely the differences between DGCD-13 and DGCD-6 in the S1 Appendix Section S1.1: “Here, we provide a brief introduction to the directed graphlet correlation distance 6 (DGCD-6). While the same calculations are performed for DGCD-13, note that all graphlets with three or fewer nodes (i.e., all 6 graphlets and 13 orbits in Figure S1) are used when employing DGCD-13, instead of the 3 graphlets (*G_0_*, *G_2_*, *G_3_*) and 6 orbits (0, 1, 5, 6, 7, 8) when employing DGCD-6. We chose to present DGCD-6 as an example derivation as opposed to DGCD-13 to simplify derivation Figures S2, S3, and S4 in Section S1.1. Specifically, the only difference between the two methods is that using DGCD-13 with bipartite networks results in (*i*) additional zero entries in orbits 2, 3, 4, 9, 10, 11, and 12 for the directed graphlet degree vector in Figure S2, (*ii*) additional corresponding zero vectors for orbits 2, 3, 4, 9, 11, and 12 in Figure S3, and (*iii*) similar to (*ii*) additional corresponding zero vectors for orbits 2, 3, 4, 9, 11, and 12 for the two directed graphlet correlation matrices, as well as additional squared terms when evaluating the DGCD equation in Figure S4.” We stress that while the additional zero entries for these orbits do allow for a more resolved multidimensional scaling figure (i.e., Figure 4 vs. Figure S7), using either method does not significantly influence our two main conclusions:

(i) both DGCD-13 and DGCD-6 find that species interaction networks are much more topologically heterogeneous compared to the next most topologically heterogeneous non-ecological bipartite networks tested. Specifically, for DGCD-13 and DGCD-6, species interaction networks are about 1.91 times and 1.76 times, respectively, more topologically heterogeneous than the next most topologically heterogeneous networks.
(ii) both DGCD-13 and DGCD-6 find that species interaction networks each sourced from a unique publication are much more topologically heterogeneous than networks sourced from the same publication. Specifically, for DGCD-13 and DGCD-6, species interaction networks each sourced from a unique publication are about 2.09 times and 1.92 times, respectively, more topologically heterogeneous than networks that share the same publication source.

2) Fig. S2: the number of times node A occurs at orbital position "0" is 3, but I think it should be 1, right?

The number of times node A occupies orbit position 0 is in fact three in this example. In order to clarify this for readers, we have now added an additional panel to Figure S2 to show how this was counted.

**Reviewer #3:** [identifies himself as Adrian David González Chaves]

Thank you for addressing all the comments. I'm pleased with your edits and enjoyed reviewing this manuscript. I learned something new in the process.

We thank Dr. Adrian David González Chaves for previous comments on the manuscript. We are also glad that something new was learned!

**Reviewer #4:**

I thank the authors for considering my comments and suggestions in this revised version of their manuscript and for producing such a detailed and nice rebuttal letter.  Thank you for the work!

We thank the Reviewer for previous comments on the manuscript. We are glad that our lengthy rebuttal was appreciated!
